# Exploring the anti-inflammatory effects of phytochemicals in attenuating interstitial cystitis-a literature review

**DOI:** 10.3389/fphar.2025.1483548

**Published:** 2025-02-05

**Authors:** Irfan Anjum, Ayesha Nasir, Faiza Naseer, Ahsan Ibrahim, Bisma Rehman, Fawad Bashir, Qura Tul Ain

**Affiliations:** ^1^ Department of Basic Medical Sciences, Shifa College of Pharmaceutical Sciences, Shifa Tameer-e-Millat University, Islamabad, Pakistan; ^2^ Department of Biosciences, Shifa Tameer e Millat University, Islamabad, Pakistan; ^3^ Shifa College of Medicine, Shifa Tameer-e-Millat University, Islamabad, Pakistan

**Keywords:** interstitial cystitis, phytochemicals, uroprotective, inflammation, inflamed bladder tissue, painful bladder syndrome

## Abstract

Interstitial cystitis is a fierce syndrome affecting the quality of life of thousands of individuals around the globe. It causes immense pain in the bladder and associated viscera along with inflammation-like lesions. The current medicinal and pharmacological research focuses on the protective and curative effects of phytochemicals in several ailments. Phytochemicals derived from many medicinal plants have shown potent outcomes in protection against various pathological conditions including interstitial cystitis. This review has summarized the insights of *in vitro* and *in vivo* studies regarding the effects of phytochemicals in fading the inflammation in bladder tissue and exhibiting a protective effect on the urothelium. Hemorrhagic cystitis is a common manifestation in patients undergoing chemotherapy with cyclophosphamide and related alkylating agents. Sodium 2-mercaptoethane sulfonate (Mesna) has traditionally been employed in clinical practice to counter cyclophosphamide-induced cystitis in humans. However, cyclophosphamide has been employed in developing animal models of interstitial cystitis in *in vivo* studies. Phytochemicals including quercetin, beta-caryophyllene, curcumol, boswellic acid, caftaric acid, some flavonoids and other secondary metabolites being a consequential component of numerous medicinal plants, have displayed a significant reduction in the levels of proinflammatory cytokines including TNF-α, NFĸB, IL-1β, NLRP3 inflammasome, IL-6, IL-2, matrix metalloproteinases etc. Uroprotective outcomes of these phytochemicals have been found to result in diminished oxidative stress and restoration of glutathione, superoxide dismutase, and related proteins in the inflamed bladder tissue. Many *in vivo* studies involving cyclophosphamide-induced interstitial cystitis have confirmed these findings. The coupling of phytotherapy with novel drug delivery systems such as nanoparticles, liposomes, nanotubes, quantum dots, etc. can help translate these beneficial effects of phytochemicals into clinical practice. Further investigations of these phytochemicals can provide intuition regarding the development of newer drug molecules having exclusive activity for attenuating interstitial cystitis.

## Introduction

**FIGURE 1:**
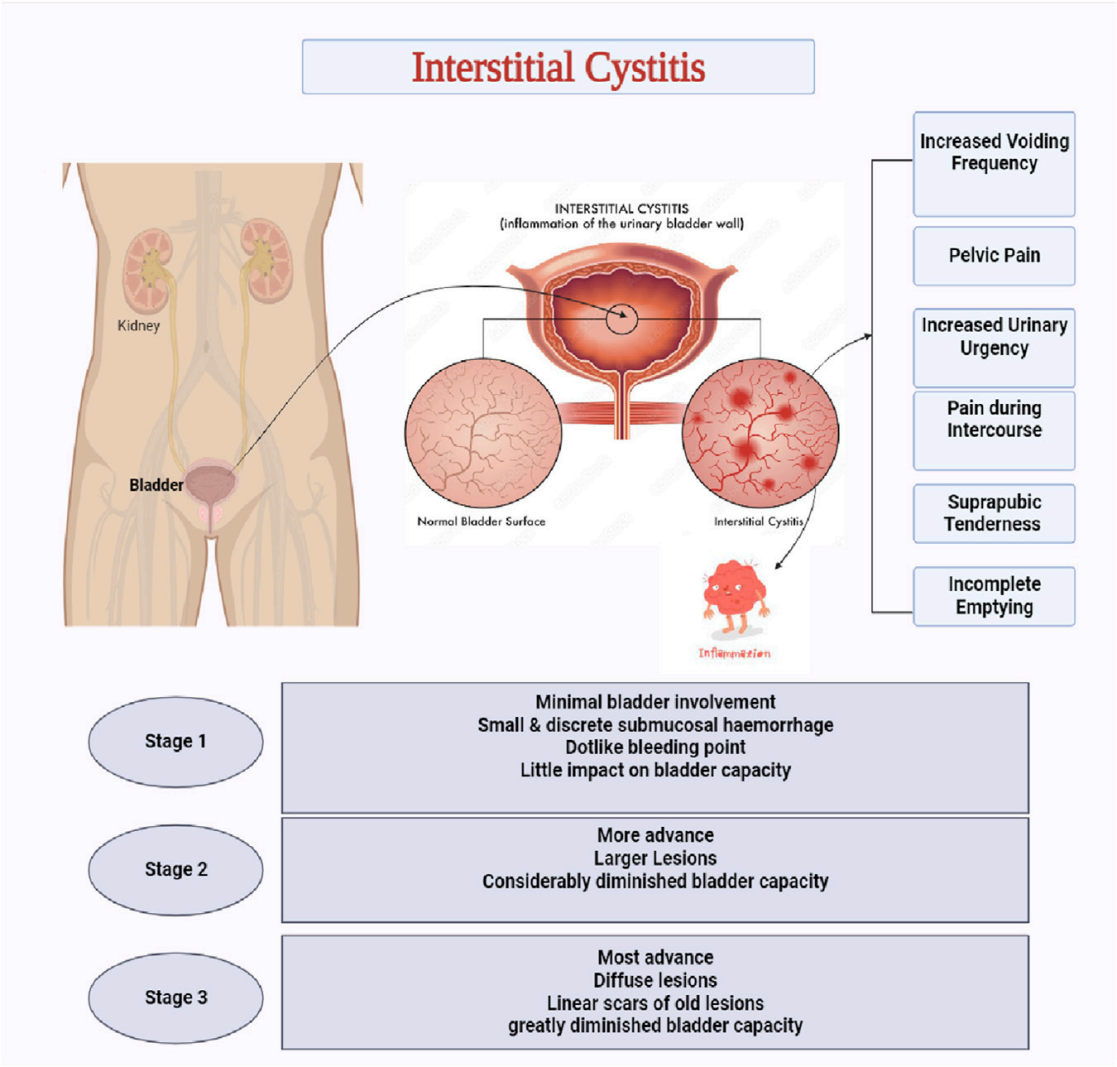
Interstitial Cystitis (Clinical manifestations and staging)

Interstitial cystitis/Painful bladder syndrome (IC/PBS) is chronic discomfort of the bladder wall, presenting with symptoms such as urinary frequency, urgency, and severe pelvic or suprapubic pain. Although the etiology is not well understood, many theories have elaborated on the probable involvement of inflammation. Unlike urinary tract infections, IC does not involve bacterial infection, as urine cultures are negative and antibiotics are ineffective. The exact causes of IC/PBS are still a topic of research. IC/PBS is categorized into Hunner-type IC (HIC), marked by Hunner lesions and severe bladder inflammation and non-Hunner IC (NHIC), where symptoms occur without these lesions and show minimal bladder pathology (Homma et al., 2020; Yoshimura et al., 2014). The pain may be mild or severe and may be described as pressure, burning, or stabbing. IC/PBS is thought to be caused by a combination of factors, including inflammatory appearing lesions, nerve damage and defects in the bladder lining (Hanno et al., 2011; Driscoll and Teichman, 2001). Diagnosis of IC/PBS involves ruling out other conditions with similar symptoms, such as urinary tract infections, bladder stones, and overactive bladder (MacDiarmid and Sand, 2007). It is a chronic condition that can significantly impair the patient’s quality of life, including their ability to work, maintain social relationships, and engage in sexual activity. In recent years, the term “painful bladder syndrome” has been added to the nomenclature to encompass a broader range of patients with this symptom complex (Abrams et al., 2010; Hanno et al., 2005). The clinical manifestation has been demonstrated in Figure 1.

IC is a chronic inflammatory condition of the urinary bladder with poorly understood etiology. It is often difficult to diagnose, especially in mild or moderate cases, as the symptoms can overlap with other gynecologic and urologic conditions, such as overactive bladder (OAB). OAB is characterized by urinary urgency, with or without urge urinary incontinence (UUI), usually with increased frequency and nocturia. In the absence of obvious pathology to account for these symptoms, IC should be considered in the differential diagnosis (MacDiarmid and Sand, 2007; Hanno et al., 2005). Table 1 depicts various phytochemicals that have demonstrated pharmacological activity against interstitial cystitis.

**TABLE 1:** Phytochemicals for the treatment of interstitial cystitis.

Plants	Phytochemicals	Molecular formula	PubChem ID	Animal model	Route of administration	Pharmacological activity	References
*Cannabis sativa*	Beta-Caryophyllene	C_15_H_24_	5281515	Mice (LPS-induced IC model)	Intraperitoneal (IP), Intravesicle, Oral	Reduction in leukocyte infiltration in bladder tissue. Pain relief. Comparable effects to a known CB2R agonist	Serra et al. 2022, Rom and Persidsky 2013, Berger et al. (2019), Cabral et al. (2015)
*Curcuma longa*	Curcumol	C_15_H_24_O_2_	14240392	Female Mice	Oral	Protective effects in interstitial cystitis. Reduction in TNF-α, IL-1β, and LD levels. Suppression of PTK2	Naseer et al. (2015), Wu et al. (2019a), Murphy et al. (2019)
*Tanacetum parthenium*	Lactone Parthenolide	C_15_H_20_O_3_	7251185	Rats	Subcutaneous	Anti-inflammatory activity. Reduction in NFκB expression and subsequent Cox-2 production	Kiuchi et al. (2024)
*Citrus depressa*	Nobiletin and Tangeretin	C_21_H_22_O_8_ and C_20_H_20_O_7_	72344 and 68077	Female C57BL/6 Mice	Intraperitoneal (IP)	Protective effects in IC mice model. Suppression of Cx43 expression. Inhibition of NFκB pathway, cytokine release, and NLRP3 inflammasome by suppressing PAMPs and DAMPs signaling. Activation of NLRP3 inflammasome by TLR activation, NFκB, PAMPs, and DAMPs. Production of IL-1β	Jo et al. (2016), Kono et al. (2022)
*Coffea arabica*	Chlorogenic Acid	C_16_H_18_O_9_	1794427	Sprague-Dawley Rats	Not specified	Anti-inflammatory effects. Maintenance of Bcl-2 expression. Reduction in pro-apoptotic proteins (Bax, caspases). Downregulation of NFκB pathway	Luo et al. (2020)
*Houttuynia cordata*	Quercetin and Hyperoside	C_15_H_10_O_7_ and C_21_H_20_O_12_	5280343 and 5281643	Female Rats	Intraperitoneal (IP)	Anti-inflammatory effects. Lower levels of pro-inflammatory cytokines (TNF-α). Increased pain threshold. Reduced immune cell infiltration and absence of hemorrhages in bladder tissue	Li et al. (2020)
*Matricaria recutita*	Apigenin	C_15_H_10_O_5_	5280443	Female Sprague−Dawley Rats	Intraperitoneal (IP) + Oral	Uroprotective and anti-inflammatory effects. Reduction in pro-inflammatory cytokine mRNA levels (TGF-β, TNF-α, IL-6). Intact bladder tissue. Molecular docking suggests TNF-α receptor inhibition	Anjum et al., (2023a), Fatima et al. (2022)
*Aster tataricus*	Shionone	C_30_H_50_O	12315507	Female Sprague-Dawley Rats	Not specified	Inhibition of NFκB pathway. Decreased expression of NFκB and NLRP3 inflammasome. Reduction in IL-1β, caspases, NFκB, and gasdermin D protein	Wang Fang et al. (2016), Wang et al. (2021)
*Abelmoschus manihot*	Isoquercetin and Rutin	C_21_H_20_O_12_ and C_27_H_30_O_16_	5280804 and 5280805	Mice (LPS-induced IC model)	Not specified	Protective activity in interstitial cystitis. Downregulation of TLR4, IL-6, IL-1β, NF-κB, and other pro-inflammatory signaling molecules	Su et al. (2022)
*Anagallis arvensis*	4-ethyl-5-octyl-2,2 bis (trifluoromethyl)–1,3-dioxalane and benzene dicarboxylic acid	C_15_H_24_F_6_O_2_ and C_27_H_22_O_9_	550234 and 6452710	Wistar Rats	Oral (Alcoholic extract) + Intraperitoneal (IP)	Protective effects in interstitial cystitis. Suppression of pain, reduction in inflammatory proteins (TNF-α, IL-6, glutathione peroxide, nitric oxide), and preservation of bladder tissue	Shabbir et al. (2022)
*Eucalyptus phellandra, Eucalyptus staigeriana, Schinus molle, Ligusticum marginatum*	α-Phellandrene	C_10_H_16_	7460	Mice (Ifosfamide-induced IC model)	Not specified	Reduction in pro-inflammatory cytokines (TNF-α), retention of glutathione levels	Gonçalves et al. (2020), Gilles et al. (2010)
*Ficus carica*	Caftaric Acid	C_13_H_12_O_9_	6440397	Female rats (Cyclophosphamide-induced IC model)	Not specified	Reversal of cystitis, reduction in pro-inflammatory cytokines, restoration of glutathione and superoxide dismutase levels, histopathological improvement	Anjum et al. (2023a), Vendramin et al. (2021)
*Uncaria tomentosa*	Quinovic acid	C_30_H_46_O_5_	120678	Mouse	Intraperitoneal	Downregulation of interleukins-beta, P2X7R expression and also the neutrophil migration inhibition	Dietrich et al. (2015)
*Potentilla chinensis*	Trans-tiliroside	C_30_H_26_O_13_	5320686	Rat	Oral	Potentilla chinensis’s mechanism of action is complicated, it is believed to have effect on the afferent and efferent urinary bladder	Juszczak et al. (2022)
*Brassica campestris*	Campesterol	C_28_H_48_O	173183	Rat	Not specified	Pharmacological activity shown on the muscuranic receptors, Ca + channels, potassium channels and COX ppathways	Javed et al. (2023)
*Silybum marianum*	Silymarin	C_25_H_22_O_10_	5213	Rats	Oral	May be antimuscurnic but exact mechanism of action is unknown	Eser et al. (2012)
*Curcuma longa*	Curcumin	C_21_H_20_O_6_	969516	Rats	Intraperitoneal	Release of TNF- alpha, NO leading to the improvement of energy levels and maintaining the normal antioxidant activity	Arafa (2009)
*Berberis vulgaris*	Berberine	C_20_H_18_NO_4_^+^	2353	Rats	Intraperitoneal	Berberine has a protective effect against hemorrhagic cystitis by reducing nitric oxide metabolites levles	Xu and Malavé (2001)
*Epimedium koreanum*	Icariin	C_33_H_40_O_15_	5318997	Mice	Intraperitoneal	Antioxidant activity showed by the increase in the levels of glutathione, superoxide dismutase, catalase Decrease in the levels of malondialdehyde, NO and myeloperoxidase activity Decrease in the production of interleukins-beta and tumor necrotic factor	Amanat et al. (2022)
Acacia senegel	*Gum acacia*	C_12_H_36_	91333377	Rats	Oral	decrease in the lipid peroxidation increase in the levels of sulfhydryl (GSH) decrease in the nitric oxide content leading to the decrease in the reactive oxidative stress increase contractility against acetylcholine	Al-Yahya et al. (2009)
*Allium sativum*	Diallyl disulfide	C_6_H_10_S_2_	16590	Rats	Gavage	Decrease in nitric oxide production and COX2 expression in bladder Downregulation of MAPK and NF-kB pathways Increase in the antioxidant activity by inhibiting lipid peroxidation Decreased levels of malondialdehyde	Kim et al. (2015)
*Viscum album*	Quercetin	C_15_H_10_O_7_	5280343	Mice	Oral	Decreasing oxidative stress by reducing superoxide levels, glutathione levels, catalases, nitric oxide levels	Sekeroğlu et al. (2011)
*Olea europaea*	Oleuropein	C_25_H_32_O_13_	5281544	Rats	Oral	Decrease in glutathione Reduced levels of NO, TNF- alpha, endothelial growth factor, its antioxidant and anti- inflammatory effect proves to provide a uroprotective effect	Sherif et al. (2016)
*Zingiber officinale*	Gingerol	C_17_H_26_O_4_	442793	Mice	Oral	Gingerol showed its antioxidant and anti inflammatory effect by activation of interleukin 10 resulting in the activation of various signaling pathways such as JAK, STAT and FOXO	Ferreira et al. (2023)
*Veratrum grandiflorum*	Resveratrol	C_14_H_12_O_3_	445154	Rats	Intraperitoneal	Increases glutathione levels Improves antioxidant activity through increasing CAT, SOD levels Increases IL-10 Decreases TNF-alpha	Keles et al. (2014)
*Centella asiatica*	Asiatic acid	C_30_H_48_O_5_	119034	Rats	Oral	Asiatic acid restored concentration of brain derived neurotropic factors, interleukin 1, and interleukin 6, tumor necrotic factor alpha. Moreover, decreasing urothelium thickness and edema of bladder. Thus, it provides an effective approach to the treatment of cystitis	Wróbel et al. (2021)
*Cuminum cyminum*	α-pinene and β-pinene	C_10_H_16_	6654 and 14896	Rats	Intraperitoneal	Decreased the hemorrhage, NO, IL-6 and tumor necrotic factor-alpha Increase in the antioxidant activity through various processes such as CAT, GPx	Anjum et al. (2023b)
*Justicia adhatoda*	Ambroxol	C_13_H_18_Br_2_N_2_O	2132	Mice	Interperitoneal	Reduction in the levels of malondialdehyde, tumor necrotic factor and glutathione	Barut et al. (2019)
*Egletes viscosa*	Ternatin	C_19_H_18_O_8_	5459184	Rats	Intraperitoneal	Results in complete blockade of hemorrhagic cystitis	Vieira et al. (2004)
*Phyllanthus niruri*	Quercetin, Gallic acid and Rutin	C_15_H_10_O_7_, C_7_H_6_O_5_ and C_27_H_30_O_16_	5280343, 370 and 5280805	Mice	Oral	Effective in treating visceral pain Decreasing the inflammation by interrupting the activation of NF-kB, AP-1 inflammatory pathway, NO release, cytokines production Decrease in the lipid peroxidation	Boeira et al. (2011)
Pterocarpus marsupium	Pterostilbene	C_16_H_16_O_3_	5281727	Rats	Oral	Decrease malondialdehyde levels, oxidative stress, apoptosis occurring in bladder	Kerimoğlu et al. (2023)
*Nigella sativa*	Thymoquinone	C_10_H_12_O_2_	10281	Mice	Interaperitoneal	Remarkable reduction in the lipid peroxidation, increasing glutathione levels, increasing the catalase activity also the enhancement in the activity of superoxide dismutase enzyme Protection against oxidation causing DNA damage Enhanced regulation of NrF2 (nuclear factor erythroid 2- related factor 2); improving bladder health	Gore et al. (2016)
*Apium graveolens*	Luteolin-glycosides, β-pinene and β-phellendrene	C_21_H_20_O_11_, C_10_H_16_ and C_10_H_16_	5319116, 14896 and 11142	Rabbit	Oral	Manuka honey enhances antioxidant activity through SOD and the CAT enzymes Reduces levels of IL-1, IL-6. TNF, NF- κB Provide better urothelial protection than celery seeds	Mousa et al. (2022)
*Glycyrrhiza glabra*	Glycyrrhizin	C_42_H_62_O_16_	14982	Rats	Gastric gavage	Alleviation of edema, inflammation, congestion, hemorrhages Protective against bladder lesions	Erdoğan and Keleş (2019)
*Caesalpinia pyramidalis*	Caesalflavone, Methyl gallate	C_30_H_20_O_10_, C_8_H_8_O_5_	5315272, 7428	Rats	Oral	Decreases myeloperoxidase activity resulting in reduced neutrophil count and inflammation Reduced malondialdehyde formation; indicating reduced oxidative stress Decrease in the serum Nitric oxide levels	Moraes et al. (2013)
*Quercus infectoria*	Isopropyl gallate	C_10_H_12_O_5_	70826	Mice	Oral	Isopropyl gallate exerts its antioxidant and anti-inflammatory activity by reducing the inflammatory hallmarks such as IL-1, TNF-alpha, MDA and also the CRP (c-reactive protein) Increases SOD (Superoxide dismutase)	Almeida de Oliveira et al. (2022)
*Mandevilla velutina*	MV8608 (newly discovered compound)	Not known yet	Not Applicable	Rats	Intraperitoneal	MV8608 Decreases the hemorrhage formation, reduces bladder weight, reduces MPO (myeloperoxidase) activity; an indicator for the migration of neutrophils They have antinoiceptive effects as well	Santos et al. (2010)
*Solanum lycopersicum*	Lycopene	C_40_H_56_	446925	Rats	Intraperitoneal	Lycopene react with singlet oxygen forming lycopene endoperoxide, hence neutralizes reactive oxygen species, preventing lipid peroxidation through interaction with peroxyl radicals Reduces maondialdehyde, increases GSH. Inhibits inflammatory pathways and enzymes such as NF-Kb, COX2	Jamshidzadeh et al. (2009)
*Punica granatum*	Ellagic acid	C_14_H_6_O_8_	5281855	Rats	Oral	Decrease in malondialdehyde levels; leading to the decrease in the oxidative stress Increase in the catalase activity and protein thiol levels; resulting in the increase antioxidant activity Anti-inflammatory effect Maintains structural integrity of bladder exhibiting lesser collagen damage Tunel assay indicating decrease in the apoptosis	Mahmoudi et al. (2018)
*Moringa oleifera*	Quercetin, Apigenin, Kaempferol	C_15_H_10_O_7_, C_15_H_10_O_5_, C_15_H_10_O_6_	5280343, 5280443, 5280863	Rats	Oral	Decrease in the malodialdehyde levels Increase in the glutathione levels	Taha et al. (2015)
*Spirulina-Arthrospira platensis*	C-phycocyanin, β-carotene, Vitamin E	C_33_H_38_N_4_O_6_, C_40_H_56_, C_31_H_52_O_3_	11606751, 5280489, 86472	Rats	Oral	Results in decrease in the levels of malondialdehyde Increase in the levels of CAT and SOD.	Sinanoglu et al. (2012)
*Ipomoea obscura*	Ipobscurine	C_29_H_28_N_2_O_7_	25235391	Mice	Intraperitoneal	Increase in the GSH levels, interferons-gamma, interleukins-2 levels Decrease in the tumor necrotic factor	Hamsa and Kuttan (2011)

HIC is a specific form of IC/BPS distinguished by the presence of Hunner lesions-areas of inflammation on the bladder lining. This subtype is characterized by intense bladder wall inflammation, including infiltration by lymphoplasmacytic cells, epithelial damage and an increase in mast cells beneath the epithelium. These cellular changes contribute to symptoms such as persistent pelvic pain, urinary urgency and frequent urination (Lin et al., 2021). From a pathophysiological perspective, HIC exhibits decreased levels of anti-inflammatory and immune-regulating factors in affected tissues. This is accompanied by a shift in macrophage populations, with a higher number of pro-inflammatory M1 macrophages and reduced anti-inflammatory M2 macrophages in Hunner lesions. This imbalance is thought to perpetuate chronic inflammation and tissue injury, setting HIC apart from other types of IC/BPS. These insights suggest potential parallels between HIC and autoimmune diseases, underscoring the need for therapies targeting inflammation and immune pathways (Maeda et al., 2015).

On the other hand, NHIC, a subtype of bladder pain syndrome (BPS), involves chronic bladder pain, frequent urination, and urgency, but lacks the observable Hunner lesions found in Hunner-type IC (Mostafa et al., 2024). Pathologically, NHIC is different in that it does not show the intense inflammatory changes seen in Hunner lesions. Instead, it is marked by mild urothelial dysfunction, where the protective layer of the bladder may be weakened, leading to heightened sensitivity to urinary irritants. This dysfunction can activate sensory nerves in the bladder, which is believed to contribute to the pain and other symptoms. While inflammation is present, it is typically less severe than in Hunner-type IC, and patients may experience mild inflammation or an increase in mast cells, which contribute to discomfort. Neural mechanisms, such as neurogenic inflammation, are also important in NHIC, where nerve activation exacerbates bladder pain. Treatments for NHIC typically aim at protecting the bladder lining and modulating nerve activity to reduce pain and discomfort (Jhang et al., 2022).

Recent advances in understanding the pathophysiology of IC/BPS have led to the development of novel treatments, which are currently being tested. The classification of IC/BPS patients based on their clinical characteristics, urodynamic findings, and urinary biomarkers may help to guide individualized therapy. The standard treatment algorithm for IC/BPS begins with lifestyle modification, followed by bladder-directed therapy. In patients with high anxiety, a combination of bladder therapy and psychological intervention may be most effective. Psychological stress is often associated with IC symptom exacerbation, such as bladder pain, urinary frequency, and urgency. In recent decades, the following treatments for IC/BPS have been recommended as replenishment of the glycosaminoglycan layer to control urothelial defects, administration of amitriptyline or imipramine to inhibit neurological hyperactivity, suppression of allergies by antihistamines, and non-steroid anti-inflammatory drugs for pain control (Homma et al., 2020).

Intravesical treatments with heparin, hyaluronic acid, chondroitin sulfate, *bacillus* Calmette-Guerin, dimethylsulfoxide, and resiniferatoxin have been effective in treating IC/BPS, but the effects were not durable (Hanno et al., 2015).

Despite the availability of multiple treatment options, which offer a wide range of possible combinations and a high level of personalization, identifying the optimal treatment for complex diseases remains challenging. This is due to the limited understanding of the underlying molecular mechanisms and disease etiology. As a result, most available treatments have a limited impact and are primarily aimed at symptom management.

Given the side effects of IC medications and the invasive nature of the treatments, it should come as no surprise that there is a significant interest in alternative and herbal therapies for IC/PBS. The World Health Organization estimates that 80% of the world’s population presently uses herbal medicine for some aspect of primary healthcare (Chughtai et al., 2013). The women are more likely than men to use complementary and alternative medicine (Simaan, 2009; Xutian et al., 2009).

This comprehensive review aims to delve into the intricate landscape of IC and assess the recent advancements in the use of phytochemicals as therapeutic agents. Phytochemicals are bioactive compounds found in plants, known for their diverse pharmacological effects, including anti-inflammatory, antioxidant, and antispasmodic activities. In recent years, researchers have explored their potential in alleviating the symptoms of IC and improving patient health. This review will critically examine the recent research studies on various phytochemicals, such as flavonoids, polyphenols, and herbal extracts, to determine their effectiveness in mitigating IC symptoms. It is imperative to explore the safety profile and tolerability of phytochemical interventions, especially in comparison to traditional pharmacological treatments. By exploring the potential of phytochemical interventions for IC, this review aspires to contribute to the literature on alternative treatments and provides a valuable resource for healthcare professionals and researchers seeking innovative approaches to improve the health conditions of patients grappling with IC.

## Epidemiology of interstitial cystitis

The prevalence of IC/PBS exhibits considerable variation globally, influenced by differences in diagnostic criteria and research methodologies. Estimates suggest a prevalence ranging from 0.01% to 6.5%, with significant regional and ethnic disparities. In the United States, studies indicate rates among women between 2.7% and 6.5%. European data show prevalence rates of approximately 300 cases per 100,000 individuals in Finland, 206 per 100,000 in Austria and 147 per 100,000 in Boston. In Asia, Korea reports around 0.26% prevalence among women, while Japan records about 1% of the general population experiencing IC/PBS. The prevalence data in Taiwan show an increase in prevalence from 21.8 per 100,000 in 2002 to 40.2 per 100,000 in 2013, while in China, the rates are relatively lower, estimated at 21.8–100 cases per 100,000 (Li et al., 2022; Homma et al., 2020).

## Pathophysiology of interstitial cystitis

IC exhibit a range of pathologies across different cell types, including those in the epithelial, endothelial, and smooth muscle (detrusor) layers, as well as in neuronal and immune cells (Phatak and Foster, 2006). The histological characteristics of IC, including the thinning and erosion of the bladder epithelium, along with heightened infiltration by mast cells, suggest that the condition may stem from the compromised regenerative capacity of the bladder’s epithelial cells, coupled with anomalies in immune system functioning (Slobodov et al., 2004). Furthermore, the urothelium also shows reduced cell growth and heightened cell permeability (Fiehn and Kim, 2014). At the molecular level, these observations are corroborated by the disrupted regulation of proteins that promote cell proliferation, such as cyclin D1, and those that form barrier-tight junctions, including zonula occludens-1, occludin, and claudins 1, 4, and 8 (Birder, 2019). In the bladder endothelium of individuals with IC, there is often a higher expression of vascular endothelial growth factor, substance P, and platelet-derived endothelial cell growth factor compared to healthy controls (Kiuchi et al., 2009). Specifically, bladder epithelial cells IC show inherent changes in differentiation, the release of neurotransmitters, and the activity of potassium channels. Additionally, there is an upsurge in nitric oxide synthesis, activation of nuclear factor-κB, an increase in nerve fiber density, elevated levels of serum C-reactive protein, and enhanced production of neuropeptide Y and nerve growth factor. This indicates that the urinary bladder is regulated by neural mechanisms (Fiehn and Kim, 2014). The development of IC is attributed to several principal factors, including.

### Epithelial dysfunction

The urinary bladder’s lining includes a bladder surface mucin that is impermeable and made up of sulfonated glycosaminoglycans (GAGs) and glycoproteins. Alterations in this protective layer may lead to changes in permeability, permitting potassium ions to pass through the urothelium. This can result in the depolarization of sensory and motor nerves and the activation of mast cells. Such dysfunction in permeability is evidenced by heightened urea uptake and positive responses to potassium sensitivity tests in patients with IC (Sant, 2002). GAGs are a group of polysaccharide molecules, also known as mucopolysaccharides, that create the foundational structure of the extracellular matrix (ECM), together with other components such as collagen, elastin, fibronectin, and laminin Biomechanically, the layer of GAGs functions as a “hydrated gel” that provides cushioning and resists compressive forces. Along with other components like collagen and fibronectin, GAGs play a role in the pathophysiological processes at different stages of the disease’s progression (Richter et al., 2010) (Treutlein et al., 2012). The urinary antiproliferative factor (APF), which has been recently identified in the context of IC, acts to hinder cell growth and disrupts the repair of the damaged or worn-away urothelium, leading to alterations in the barrier function of the bladder lining (Sant, 2002).

### Inflammatory mediators

Any disturbance in tissue equilibrium and the cellular milieu can prompt the release of chemical signals or trigger responses. These changes initiate the activation of a particular group of immune cells known as mast cells (MCs). Mast cells are mainly found in the bone marrow and play a role in innate immunity as well as in neurogenic and autoimmune responses (Sant et al., 2007; Metz and Maurer, 2007). (Meijlink, 2014; Hanno et al., 2011). MCs release a variety of biologically active substances, including histamine, heparin, serotonin, kinins, proteases, phospholipases, chemotactic factors, cytokines, and vasoactive intestinal peptides. Additionally, they can synthesize molecules like interleukin-6 (IL-6), leukotrienes, platelet-activating factor, prostaglandins, thromboxane, nitric oxide (NO), and tumor necrosis factor-alpha (TNFα) from scratch (Peeker et al., 2003). Various agents such as anaphylatoxins, antigens, bradykinin, cytokines/lymphokines, hormones, immunoglobulin E (IgE), neurotransmitters, neuropeptides, viruses, bacterial toxins, medications, and stress can activate macrophages (Tsai et al., 2010). The ulcerative variant of IC/PBS is characterized by a more significant presence of MCs, elevated levels of histamine, and increased production of nitric oxide synthase (NOS) compared to the non-ulcerative form (Logadottir et al., 2013). The non-ulcerative type of IC/BPS involves dysfunction of the urothelium and an increase in sensory nerve activity, which occurs both in the peripheral and central nervous systems (Parsons, 2007). The nerve growth factor (NGF) is synthesized by both the urothelium and smooth muscle in the urinary tract. Research has demonstrated that when painful and inflammatory conditions affect the lower urinary tract, there is an elevation in the levels of NGF in both the bladder tissue and urine (Naseer, 2014).

Another significant factor in IC is the NF-kB pathway, which is activated by inflammatory stimuli. NF-kB plays a central role in controlling immune responses, but its overactivation in IC leads to increased expression of inflammatory mediators and a reduction in normal cell turnover. This contributes to a failure in healing, as the urothelium is unable to regenerate efficiently due to the dominance of inflammatory signals. Additionally, TGF-β (transforming growth factor-beta) signaling is disrupted, which normally regulates cell growth and repair. In IC, TGF-β fails to effectively mediate tissue regeneration, leading to fibroblast activation and fibrosis, which further inhibits cell proliferation (Mormone et al., 2024).

### Microbial/infection

Many specialists have considered IC/PBS to possibly have an infectious origin (bacterial, viral, or fungal) because microorganisms like *Helicobacter pylori* have been found in association with the condition, which draws parallels to chronic gastritis (Atuğ et al., 2004; Winnard et al., 2006). A recent study has proposed that newly identified pathogenic agents, referred to as Nanobacteria (NB), may play a role in the development of IC/PBS in certain individuals (Zhang et al., 2010). Further research is needed to clarify the potential pathological connection between NB and the symptoms or emergence of IC/PBS. In various cases of IC/PBS thought to be related to pathogens, additional analyses such as polymerase chain reactions, electron microscopy, and antibody detection have been performed. These studies have often led to the conclusion that the presence of microorganisms is a false positive reaction rather than a contributing factor to the onset of IC/PBS (Atuğ et al., 2004; Zhang et al., 2010). Upon synthesizing global research findings, experts reached a consensus to exclude lower urinary tract infections as a symptom or diagnostic criterion for IC/PBS (Hanno and Dmochowski, 2009).

### Neural upregulation

Neuroendocrine pathways, such as the sympathetic nervous system and the hypothalamus-pituitary-adrenal (HPA) axis, oversee the body’s inflammatory responses. Cell signaling is crucial for communication between organs and the nervous system, as well as between the brain and endocrine system. In many patients with IC/PBS, these signaling pathways are found to be either dysfunctional or interrupted at some point during the disease’s progression (Hanno and Dmochowski, 2009; Keay, 2008).

FIC, which is broadly recognized as the natural animal equivalent of IC/PBS in humans, demonstrates similar pathological characteristics. In these feline models, one clinical feature that mirrors the human condition is the heightened activity of the sympathetic nervous system in connection with bladder functions (Westropp et al., 2003). Additionally, in cats with FIC, there is observed increased activity of the enzyme tyrosine hydroxylase within the locus coeruleus (LC) of the brainstem. Tyrosine hydroxylase is involved in the synthesis of catecholamines, leading to heightened production of substances like dihydroxyphenylalanine (DOPA), norepinephrine, and dihydroxyphenylglycol (DHPG) (Buffington et al., 2002). When subjected to stress, cats with FIC exhibit elevated levels of catecholamines in their urine. Likewise, higher concentrations of norepinephrine have been reported in patients with IC. This area warrants additional research for a deeper understanding.

### Cholinergic anti-inflammatory pathways

Neurotransmitters, specifically adenosine triphosphate (ATP) and acetylcholine (ACh), are responsible for mediating the activation or initiation of contractions in the urinary bladder’s smooth muscle through the engagement of purinergic and/or muscarinic receptors (Tsai et al., 2012). (Driscoll and Teichman, 2001). In both rats induced with IC/PBS and human patients suffering from IC/PBS, there is a notably high expression of these purinergic and muscarinic receptors (Nasrin et al., 2013). In a mouse model of IC/PBS, the enzyme Ca^2+^/calmodulin-dependent protein kinase II (CaMKII) is implicated in the onset of neurogenic IC and associated pelvic pain (Yang et al., 2012). While the pain in mice models is induced by a viral agent, it is also feasible to observe parallels in humans, with instances of Ca^2+^/CaMKII protein involvement in patients with IC/PBS. FIC remains the sole spontaneous animal model that naturally reflects the IC/PBS condition (Westropp et al., 2003; Hanno et al., 2008). The study also revealed that the expression of α-smooth muscle iso-actin was relatively higher than that of γ-smooth muscle iso-actin. The smooth muscle’s structure seemed to be proliferative and migratory, and somewhat “synthetic,” indicative of a disruption in the differentiation process. These “synthetic” type smooth muscle cells, characterized by their high expression of α-smooth muscle actin, suggest that this marker may be valuable for diagnosing IC/PBS (Lutgendorf et al., 2002).

### Genetic predisposition

First-degree relatives have a higher likelihood of developing IC/PBS compared to the general population, indicating that the occurrence of the disease may have a zygotic link, whether monozygotic (identical twins) or dizygotic (fraternal twins) (Warren et al., 2004). A recent study has shown that the occurrence of IC/BPS in both monozygotic and dizygotic twins, regardless of gender, may not be solely due to genetic factors (Altman et al., 2011). Additional research is required to determine definitively whether IC/PBS has a hereditary component.

It has been established that several dietary factors can worsen the symptoms of IC/PBS. These include alcohol consumption, smoking, foods that act as stimulants, foods high in acid such as certain fruits (e.g., pineapples, oranges), foods rich in potassium, caffeinated beverages like tea and coffee, and various other dietary elements (zhong et al., 2010; Friedlander et al., 2012). Despite these observations, it is still not clear whether there is a direct causal relationship between food and the symptoms of IC/PBS.

## Molecular mechanism of interstitial cystitis

Patients diagnosed with IC exhibit a range of abnormalities within various cell types, including epithelial, endothelial, smooth muscle (detrusor), neuronal, and immune cells (Phatak and Foster, 2006). Certain histological characteristics of IC, such as the thinning of the bladder epithelium, erosion, and heightened mast cell infiltration, suggest that the condition may result from a deficiency in the regenerative capacity of bladder epithelial cells, along with immune system dysfunction (Slobodov et al., 2004). Furthermore, urothelium from individuals with IC has demonstrated reduced cell proliferation and increased cell permeability (Fiehn and Kim, 2014). These observations are corroborated at the molecular level, with dysregulation in the expression of pro-proliferative proteins (e.g., cyclin D1) and tight junction proteins (e.g., zonula occludens-1, occludin, and claudin 1, 4, and 8) (Birder, 2019). One key factor is the dysregulation of inflammatory pathways, which significantly affects the bladder’s ability to repair and regenerate. In particular, the JAK/STAT signaling pathway is frequently implicated in IC. Normally, this pathway helps regulate immune responses and cell growth, but in IC, it is often activated abnormally, leading to excessive production of inflammatory cytokines like CCL8 and VEGFD. These cytokines contribute to a pro-inflammatory environment, hindering the proliferation of urothelial cells. Chronic activation of the JAK/STAT pathway results in sustained inflammation, creating a feedback loop that prevents effective tissue repair and exacerbates urothelial damage (Peskar et al., 2023).

In interstitial cystitis (IC), significant gene expression changes disrupt normal cell proliferation and tissue repair, contributing to chronic symptoms. For instance, cyclin D1, which is essential for regulating the cell cycle, is downregulated, often due to the dysregulation of the phosphatidylinositol 3-kinase/protein kinase B (PI3K/Akt) pathway. This impairs cell proliferation and survival. E-cadherin, a cell adhesion molecule, is upregulated, disrupting tissue integrity and is influenced by the Wnt/β-catenin signaling pathway, which affects cell-cell interactions and tissue cohesion. Additionally, the downregulation of vimentin, a marker for epithelial-to-mesenchymal transition, is linked to changes in transforming growth factor (TGF)-β signaling, affecting cell differentiation and impairing regenerative processes in the bladder. Altered expression of α2-integrin, α-catenin and genes associated with the Switch/Sucrose Non-Fermentable (SWI/SNF) complex further exacerbate these issues, creating an environment where bladder epithelial cells struggle to regenerate, thereby contributing to the persistent symptoms of IC (Park et al., 2024; Jin et al., 2021).

Additionally, higher levels of vascular endothelial growth factor, substance P, and platelet-derived endothelial cell growth factor are often detected in the bladder endothelium of IC patients compared to controls Notably, bladder epithelial cells in individuals with IC exhibit intrinsic alterations in differentiation, neurotransmitter release, potassium channel activity, increased nitric oxide production, nuclear factor-κB activation, nerve fiber density, serum C-reactive protein levels, and the production of neuropeptide Y and nerve growth factor. These findings suggest that neural control plays a significant role in the functioning of the urinary bladder in IC (Fiehn and Kim, 2014).

### Phytochemicals as anti-inflammatory agents in interstitial cystitis

Medicinal plants have been used for decades to attenuate several ailments. Phytochemicals have proven anti-inflammatory, anti-cancer, anti-infective, anti-oxidant, hepatoprotective, and other therapeutic effects that help to fight many diseases (Riaz et al., 2023). The scientific literature refers to many studies that have postulated the potential anti-inflammatory effects of many phytochemicals in interstitial cystitis. A study reported the protective effects of nobiletin and tangeretin in ICin mice model. Nobiletin and tangeretin belong to the group of polymethoxyflavonoids, found in the peel extract of *Citrus depressa* (Jang et al., 2013). In the *in vivo* experiment, female C57BL/6 mice were injected with cyclophosphamide via intraperitoneal route. About 24 h prior to cyclophosphamide administration, the mice were injected with PMF90, i.e., dispersion of 90% nobiletin and 30% tangeretin in corn oil. Afterward, the urothelium of the mice was extracted and studied for the expression of inflammatory markers such as connexin 43 (Cx43) protein. Western blot analysis showed that the urothelium of mice pre-treated with PMF90 expressed small amounts of Cx43 and other inflammatory markers as compared to mice group receiving cyclophosphamide alone. In the *in vitro* experiment, hTERT immortalized human urothelial cell line was exposed to various concentrations of IL-1β. The subsequent upregulation of Cx43 was recorded. This IL-1β associated surge in Cx43 expression was diminished by exposure of the cell line to nobiletin (Kono et al., 2022). The underlying mechanisms of nobiletin and tangeretin include the suppression of signaling pathways after pathogen-associated molecular patterns (PAMPs) and damage-associated molecular patterns (DAMPs). The nuclear factor-kappa B (NFκB) pathway associated cytokine release is inhibited, hence weakening the signal transduction by IL-1β and nucleotide-binding domain, leucine-rich–containing family, pyrin domain–containing-3 (NLRP3) inflammasome (Chen et al., 2019). NFκB signaling is majorly triggered by tumor necrosis factor-alpha (TNF-α) and some other signaling molecules leading to enhanced transcription of pro-inflammatory proteins, hence playing a vital role in rendering inflammation (Biswas and Bagchi, 2016). NLRP3 inflammasomes comprised of the signaling proteins that transduce the activation of downstream inflammatory proteins. NLRP3 inflammasome is activated by toll-like receptor (TLR) activation, NFκB, or some PAMPs and DAMPs resulting in maturation and production of cytokines such as IL-1β, which is the primary inflammatory protein involved in inflammatory pathways (Jo et al., 2016).

### Abelmoschus manihot

*Abelmoschus manihot* is a medicinal plant belonging to the Malvaceae family. *Abelmoschus manihot* extracts have exhibited anti-inflammatory, anti-oxidant, anti-infective, and immunomodulatory (Indradi et al., 2018; Li et al., 2016). *Abelmoschus manihot* contains quercetin, isoquercetin, and rutin as principal phytochemicals. Isoquercetin and rutin have well-known anti-inflammatory effects. Isoquercetin has been shown to modulate the toll-like receptor 4 (TLR4) and NF-κB, while the pharmacological activity of rutin revolves around inhibition of pro-inflammatory signals such as IL-6 and IL-1β signaling (Ma et al., 2018; Cosco et al., 2016). A study developed a lipopolysaccharide (LPS) induced ICmodel in mice. The test group received a cocktail of LPS and *A*. *manihot* extract. The findings of this study ascertained that the extract of *A*. *manihot* had protective activity in interstitial cystitis. A decline in the expression of TLR4, IL-6, IL-1β, NF-κB, and many other pro-inflammatory signaling molecules, was detected using qPCR, hence demonstrating the anti-inflammatory effects of A. *manihot* in IC (Su et al., 2022).

### Acacia senegal

*Acacia senegal* is one of the most important dryland trees in sub Saharan Africa, it has economic and ecological value (Gray et al., 2013). Previous work on the extraction of cellulose has been done using *Acacia senegal* starting with bleaching and then alkali and acidic treatments (Parihar et al., 2022). Although *Acacia senegal* has been employed for different ailments such as gastrointestinal and respiratory ailments pharmacological use of *Acacia senegal* for the treatment of cystitis as specified above is remarkably scarce in the investigative documents available in the above-mentioned research papers. However the pharmacological effect of the plant also has anti-inflammatory properties that might be useful in the case of cystitis (Subhan et al., 2018; Hashim et al., 2022). The aim of this research work is to assess extent of a natural protector like Gum Arabic on antagonizing the prospective urotoxicity of CYCL in male Swiss albino rats. The latest studies indicate following CYCL administration, several changes in the bladder function can be shown by the following; There occurred a change in the following with the oxidative stress marker enzymes, Glutathione level, Isometric bladder pressure, and histology changes. In this study, the addition of GA substantially alleviated these effects and restored oxidant/antioxidant balance, bladder tone, and reducing inflammation and tissue damage. From these findings, it may be concluded that a part of the protective mechanism of GA is the detoxication of reactive metabolites including acrolein and other intermediates of oxidation stress reducing CYCL-induced damage to bladder tissues and promoting structural as well as functional betterment of the bladder and tissues (Al-Yahya et al., 2009).

### Adhatoda vasica

Furthermore, ambroxol is a mucoactive drug that possesses antioxidant activity and anti-inflammatory effects, applied for the treatment of respiratory disorders (Barut et al., 2019). It is obtained from the plant *Adhatoda vasica* and due to its reported pharmacological effects, it protects to the bladder against cystitis from CYP (Barut et al., 2019). Concerning pharmacological therapy, medicines used in the treatment of IC are those targeting the disease’s confirmed etiology, including mucin substitutes and antihistamines (Amrute and Moldwin, 2007). IC is also a long-term illness that mostly affects females and manifests symptoms such as urinary incontinence, frequency, and pelvic discomfort; these heavily influence the quality of life domain (Lukban, 2003; Gonsior et al., 2017). Generally, current treatments of IC are solely concerned with symptomatic relief of the condition, with the most effective course of treatment for the disease yet to be confirmed through continuing clinical trials designed to amplify the knowledge of the causative factors of IC, as well as to enhance the treatment regime (Lukban, 2003; Gonsior et al., 2017). AMB, 100 mg/kg, was given for 3 days before CYP-induced HC in male BALB/c mice and, thus, enhanced the frequency of acetylcholine-inducible contraction in the bladder. AMB also lowered oxidative stress and inflammation as proven by the decrease in MDA and TNF-α concentration, and avoided the suppression of total glutathione levels. Nevertheless, AMB did not affect bladder weight as well as some histological characteristics, which points to the fact that although it alleviates some effects of HC it may not prevent structural changes in the bladder (Amrute and Moldwin, 2007).

### Allium sativum

Many pharmacological effects of *Allium sativum* garlic are mainly attributed to its major compound called diallyl disulfide (DADS). Scholars have proven that DADS have exhibited anti-inflammatory effects and have free radical hunting, anti-bacterial, cardio, neuro protection and anti-carcinogenic effects (He et al., 2020; Li et al., 2023). Literature research revealed that the extraction of the DADS might be further improved using ultrasonication and non-ionic polymers (Singh et al., 2020). Relating to cystitis, the involvement of DADS has been determined in terms of precedential activity against hemorrhagic cystitis induced by cyclophosphamide in rats. DADS were identified to have diminished inflammation, oxidative stress and DNA lesions on the bladder over the topics of pro-inflammation cytokines regulation and also the MAPKs signaling pathways (Anwar et al., 2020). Furthermore, it was also proven that DADS have a renoprotective effect on methotrexate-induced nephropathy, which postures that DADS may have therapeutic value in reducing the chemotherapeutic agents’ effects like methotrexate (Nanjwade et al., 2015). The work examined the effect of DADS contrary to the cystitis on rats mediated by cyclophosphamide (CP). The present study demonstrated that CP treatment develops HC with associated histopathological changes, CNP upregulation, increased hepatic pro-inflammatory cytokines and oxidative stress, and Augmented protein levels of NF-κB, and MAPKs. DADS before surgery alleviated HC by decreasing the histopathological changes, cytokines, oxidative stress indicators, and levels of DNA damage. Also, DADS inhibited the Necrotic factor-κB, COX-2, inducible necrotic factor, and tumor necrotic factor-alpha. These findings show that diallyl disulphide can attenuate cyclophosphamide mediated by cystitis through blocking inflammation mediated by NF-κB and MAPKs pathways and the related antioxidative effect; thus, maybe provide therapeutic effect in bladder injuries (Anwar et al., 2020).

### Anagallis arvensis

*Anagallis arvensis,* a medicinal herb of the Primulaceae family contains enormous phytochemicals such as kaempferol, quercetin, saponins, anagallosides A and B, cucurbitacin B and D, sterols, etc. All of these phytochemicals contribute to the anti-inflammatory and antioxidant effects mediated by *A. arvensis* extract (Yasmeen et al., 2020). *A. arvensis* extract was used in an animal model to examine its protective effects in interstitial cystitis. Cyclophosphamide-induced IC was developed in Wistar rats. The test group was supplied with oral alcoholic extract of *A. arvensis* along with intraperitoneal doses of cyclophosphamide, while the control group revived normal saline, positive control was treated with mesna and negative control with cyclophosphamide alone. The nociceptive response was noted by putting rats in a box having nine squares and it was revealed that the *A. arvensis* extract treated group crossed more boxes showing the suppression of pain in the test group. After assessment of the isolated bladder tissue, the levels of inflammatory proteins (TNF-α, IL-6, glutathione peroxide, nitric oxide, etc.) were found to be lower in both mesna and extract-treated groups. The uroprotective effects of *A. arvensis* extract were confirmed in histopathological analysis where morphological features of the bladder epithelium of the rats were conserved in the test group. The signs of hemorrhages, edema, and tissue damage were absent in the test group (Shabbir et al., 2022).

### Apium graveolens

Another product that can be obtained from the plant and has seen its benefits in the provision of nutrients is celery seed oil, an essential phytoconstituent of *Apium graveolens* (Sharma et al., 2022). In the extraction of the oil, the natural nutritious ingredients are maintained, and the final product has anti-cancer effects and a very high mineral content (Mortada et al., 2020). Interstitial cystitis has also demonstrated that honey can in some ways help due to its possible ability to decrease histamine reactions in the bladder (Malik, 2017). Rabbits in a study that used mesna, celery seed oil, and manuka honey to develop a novel regimen demonstrated total defense against hemorrhagic cystitis caused by cyclophosphamide due to the revealed anti-inflammatory, anti-oxidant and anti-fibrotic benefits (Mousa et al., 2022). Thus, the extraction method of the celery seed oil and the potential of manuka honey to be therapeutic make them plausible approaches to treating IC because they are a combination solution for the treatment of the disease, which considers the necessity of coping with the symptoms and the improvement of the general state of the bladder. Many patients who have undergone cyclophosphamide treatment present with severe hemorrhagic cystitis. Past study suggests that the combined effect of mesna plus celery provided the partial protection of HCB stating the requirement for better (different) treatment protocols. The present investigation evaluated the efficacy of Mesna with Celery seeds oil (MCSO) and Mesna plus Manuka Honey (MMH) in HC produced by CP in male adult rabbits. Rabbits were divided into four groups and treated for about 3 weeks, control group was named G1 and administered distilled water, while group 2 received CP at a dose of 50 mg per kg per week. CP-activated HC features in G2 involving urothelial necrosis, ulceration, fibrosis, and TNF-α content being elevated while antioxidant enzyme activity is lowered, pro-inflammatory cytokines are upregulated. Signed with asterisks, the results for G4 revealed the CPMMH regimen having substantial UB protection over G3 where the CPMCSO regimen offered partial protection; *p* < 0.05. This new CPMMH regimen was protective of the UB without any CP-induced HC damage under antioxidant, anti-inflammatory, and antifibrotic actions (Mousa et al., 2022).

### Arthrospira platensis

*Arthrospira platensis* (Spirulina) is a type of blue-green algae that has significant value because many compounds with bioactivity can be synthesized that are useful in the health advantages of this organism (Eltantawy et al., 2018). These are essential and unsaturated fatty acids including GLA, linoleic acid, and oleic acid; amino acids; carotenoids especially beta-carotene, zeaxanthin, and lutein; chlorophyll; phycobiliprotein; phenolic; and vitamin and mineral (Khan et al., 2005). Spirulina contains antioxidant and anti-inflammatory, neuroprotective, anticancer, and immunomodulatory effects as has been explored (Khan et al., 2005) (Lupatini et al., 2017). Two research articles have been published on the preventive role of spirulina in animal models for CP-induced cystitis. Male rats with cystitis induced by CP at a dose of 150 mg/kg given intraperitoneally received spirulina extract at the dose of 1,000 mg/kg of p. o for 7 days before cyclophosphamide injection. Pretreatment and concomitant administration of spirulina exhibited a splendid impact on CP-elicited bladder hemorrhage, edema, and histopathological alterations possibly by way of its antioxidant and anti-apoptotic activities. Thus another study showed that pre-and post-treatment with spirulina powder (600 mg/kg, p. o) of the rats before and after injection of CP (40 mg/kg, i. p.) has protective effects against the induced oxidative stress, apoptosis, and histopathological changes in the renal and bladder tissues. Therefore, it can be concluded that spirulina in doses equal 100–500 mg/kg alleviate cyclophosphamide-induced cystitis and nephrotoxicity in animals (Eltantawy et al., 2018).

### Aster tataricus

*Aster tataricus* is a traditionally used medicinal plant containing the major phytoconstituent, a triterpenoid known as Shionone (Ma et al., 2020). The anti-inflammatory effects of shionone were studied on activated macrophages. This *in vitro* analysis manifested the upregulation of IκBα expression, resulting in the inhibition of NF-κB pathway, hence providing anti-inflammatory effects (Wang Fang et al., 2016). Another study was undertaken to test the anti-inflammatory activity of shionone in a rat model. The IC model was established in female Sprague-Dawley rats using cyclophosphamide injection, while the test group was injected with a mixture of cyclophosphamide and shionone solution. The rats were dissected, and the mRNA was collected from the bladder tissue of rats. qPCR deduced a significantly decreased expression of NFκB and NLRP3 inflammasome. An *in vitro* experiment was also a part of this study. The effects of shionone on the human uroepithelial cell line (SV-HUC-1) challenged with cyclophosphamide were also evaluated. A decrease in IL-1β, caspases, NFκB, and gasdermin D protein along with intact viability of the cells was witnessed after treatment of the cell line with shionone (Wang et al., 2021). Another study utilized the whole extract of *A. tataricus* to investigate its effects in the ICinduced pyroptosis in urothelium of urinary bladder in rat model. A decline in the levels of pro-inflammatory cytokines was noticed. The histopathological analysis showed the upheld cell viability in the group injected with extract, while increased cell lysis was found in the control group (Wang et al., 2020).

### Berberis vulgaris

The compound that attracts the most interest is **berberine**, an alkaloid that is present in plants of the Berberidaceae and Ranunculaceae family (Singh et al., 2020). *Berberis vulgaris* contains considerable levels of berberine. This has demonstrated several pharmacological properties these include; antimicrobial, antitumor, antidiabetic, and anti-inflammatory. Moreover, demethyleneberberine derived from berberine has shown appropriate pharmacokinetics and bioavailability (Li et al., 2023). Also, the role of berberine due to the preventive role the cyclophosphamide hemorrhagic cystitis i.e., the protective activity was decreased in the bladder damage and nitric oxide metabolism which proves the efficacy of berberine in the treatment of urotoxicity (Xu and Malavé, 2001). Berberis lycium which is rich in berberine has also been studied for its various cytotoxicity role on cancer cells, the compounds such as oxy berberine and β-sitosterol effectively inhibit cell proliferation and hence used in anticancer drug discovery (Anwar et al., 2020). The urotoxicity of cyclophosphamide was studied and severe hemorrhagic cystitis in rats was observed with features of bladder enlargement, the occurrence of bleeding and increased levels of NO levels in urine as well as in plasma. The effects of intraperitoneally given berberine at different doses such as 50 mg, 100 mg, or 200 mg per kg before cyclophosphamide administration can decrease CP-mediated hemorrhagic cystitis effectively in dose-dependent behavior. Two doses of Berberine were found to have better protection than a single dose, Berberine administered at 200 mg per kg or two hemi concentrations of 100 also the 200 mg per kg effectively blunted bladder oedema, hemorrhage and nitric oxide metabolite elevation. Based on these observations, it is concluded that berberine may become a potential drug in the protection and management of cyclophosphamide–provoked urotoxicity (Xu and Malavé, 2001).

### Boswellia serrata

*Boswellia serrata,* a traditional herb, belonging to Burseraceae family is being used in arthritis, diabetes mellitus type 2, and Alzheimer’s disease (Gomaa et al., 2021). Several experiments have been conducted to explore the anti-inflammatory effects of *B. serrata* extract. An *in vitro* experiment carried out the exposure of the inflamed porcine aortic endothelium with the extract of *B. serrata*, which resulted in protective effects (Bertocchi et al., 2018). A pentacyclic triperpene, boswellic acid is one of the cores phytoconstituents of *B. serrata*. Boswellic acid counters the action of cytokines involved in tissue damage due to inflammation such as IL-1, IL-2, INF-γ, and TNF-α. The inhibition of NFĸB mediated transcription of pro-inflammatory cytokines is also blocked by Boswellic acid (Ammon et al., 2016). A research study developed an ICmodel in female rats after injecting 3 doses of cyclophosphamide on 1^st^, 4^th^ and 7^th^ day of the study. The control group received saline, the negative control was injected with cyclophosphamide only, and the positive control received cyclophosphamide along with mesna while the test group received boswellic acid at concentrations of 100 and 200 mg per kg before cyclophosphamide administration. Pain thresholds were increased in boswellic and mesna-treated groups while the bladder weights were reduced up to a significant extent in the boswellic acid-treated group. The bladder tissues were rapidly extracted for further analysis. The biomarkers of oxidative stress such as superoxide dismutase and malondialdehyde were significantly lessened in the test group. The cytokine level measured through the ELISA technique reflected auspicious outcomes in terms of reduction in IL-1, IL-6, and TNF-α levels. Microscopically, the group treated with boswellic acid indicated an intact cellular framework along with no apparent signs of inflammation, showing the potent anti-inflammatory activity of boswellic acid. The same outcomes were unveiled in mesna treated group, while the bladder tissue samples of all other groups presented necrotic cells with distorted epithelial organization (Fatima et al., 2022).

### Brassica campestris

**Campesterol** is a phytosterol obtainable from plant origin i.e., *Brassica campestris* and displays a high potential pharmacological profile in the management of cystitis. The findings obtained in the course of the preceding basic research indicate that campesterol has anti-inflammatory and antioxidant activities reducing nociception, edema, and inflammatory cytokines (Javed et al., 2023). Also, Mebendazole reduces the inhibitor behavior influencing the arising of plugged hair, so appears the spasmolytic effect of campesterol in interstitial cystitis shows its therapeutic usage (Javed et al., 2023). Additionally, the interaction of campesterol with other plant extracts with phytosterols like oil of hempseeds and extract of pine bark has demonstrated an ability to decrease injuries of the urothelium, pointing to the patient’s painful symptoms from the bladder (Tafuri et al., 2023). These findings show the source of campesterol derived from plant materials, the method of extraction, and probable contribution to the management of cystitis. The present investigation was undertaken to evaluate the uroprotective effect of campesterol against interstitial cystitis which was induced chemically by employing CYP in rats. CYP with 150 mg per kg given intraperitoneally caused IC associated with changes in the oxidant-antioxidant balance and inflammation. Campeosterone at 70 mg/kg brought down the degree of nociception, edema, hemorrhage, and protein leakage significantly. It also showed an antioxidant effect in reducing the levels of MDA and NO at the same time elevating the level of superoxide dismutase, catalases, and glutathione peroxidase. It also had anti-inflammatory properties as it downregulated the levels of interleukin 1, tumor necrotic factor-α, and transforming growth factor- β. Histopathologically, maintained urothelial architecture. Thus, *in silico* studies confirmed that Campesterol has a spasmolytic effect on bladder overactivity through muscarinic receptors, VGCC, KATP channels, and COX pathways. Therefore, based on these findings efflux of campesterol can be proposed as the therapeutic option for CYP-induced IC (Javed et al., 2023).

### Caesalpinia pyramidalis

The ethanol extract of *Caesalpinia pyramidalis* (EECp) has also been considered for some pharmacological occurrence and probable therapeutic utilization. Authors of studies that investigated the pharmacological properties of EECp testing its effects on inflammation, pain, and oxidation concluded that EECp is a potential candidate for the treatment or management of many diseases (Moraes et al., 2013). Furthermore, in its bark has been determined that lupeol, acacetin phenylpropan hit acids, antioxidants and cytotoxic are responsible (Vilela et al., 2021). In addition, investigations on *Caesalpinia bonducella* have suggested that it contains anti-nociceptive, anti-diarrheal, and central nervous system depressing properties of medicinal implications (Ahmed et al., 2004). The results presented in this study showcase the therapeutic versatility of the *Caesalpinia* species with an emphasis on interstitial cystitis treatment with the help of the extracts from the Caesalpinia pyramidalis plant. According to pharmacological studies, ethanol extract of *C. pyramidalis* (EECp) could checkmate CYP-induced HC on male Wistar rats. The study has found that EECp given orally at a dose of 100–400 mg/kg reduced the MPO activity and NOx-in the inflamed urinary bladder tissues which are indicators of anti-inflammatory and anti-oxidative stress effects. While EECp failed to downsize bladder edema with the comparable efficiency of mesna, it enhanced significantly the histopathological scores and diminished myeloperoxidase activity in the lung, as well as, the levels of malondialdehyde in the bladder, lung, and spleen tissue. These findings imply that EECp decreases the volume of urinary bladder damage during cyclophosphamide-induced HC through its anti-inflammatory and antioxidant activities (Moraes et al., 2013).

### Cannabis sativa

Another study reported the anti-inflammatory potential of beta-caryophyllene, a sesquiterpene present in the *Cannabis sativa L.* plant. Beta-caryophyllene has well-pronounced antioxidant effects along with a tendency to counter inflammatory damage (Serra et al., 2022). It acts upon cannabinoid receptors 1 and 2 (CB1R and CB2R), belonging to the family of G-protein coupled receptors. CB1R mostly resides in the central nervous system (CNS) while the CB2R receptors are mostly expressed in the immune cells such as natural killer cells, neutrophils, and monocytes as well as other tissues such as the bladder, intestine, etc. Agonism at CB2R is related to the anti-inflammatory and immunomodulatory activity (Rom and Persidsky, 2013; Cabral et al., 2015). LPS-induced IC model was developed by injecting LPS through two routes. The first model involved intraperitoneal administration of LPS (the test group was provided with LPS + beta-caryophyllene through the intraperitoneal route). The second model featured the intravesicle injection of LPS in the mice (the test group received LPS + beta-caryophyllene through the intravesicle route). The third model was based on the intravesicle injection of LPS in the mice (the test group received LPS via the intravesicle route while beta-caryophyllene was administered orally). In all the beta-caryophyllene treated groups, the leukocyte infiltration was reduced in the bladder tissue of the mice, with no distinct hallmarks of inflammation. The pain was significantly relieved in the group treated with oral beta-caryophyllene. The anti-inflammatory effects of beta-caryophyllene were also compared with positive control groups treated with a known CB2R agonist. The outcomes were quite comparable. This suggests the role of beta–caryophyllene in reducing the inflammation associated with IC (Berger et al., 2019).

### Centella asiatica

**Asiatic acid**, majorly found in *Centella Asiatica* is a pentacyclic triterpene and has gained much attention due to its pharmacological effects and application (Mushtaq et al., 2023; Wróbel et al., 2021; Lv et al., 2018; Nagoor Meeran et al., 2018). The compound displays many biological has many biological actions including anti-inflammatory, antioxidant activities, neurogenic, anti-microbial, and antitumor. Asiatic acid has evidence of various prerequisites in laboratory animals in diseases such as hypertension, neurodegenerative disorders, diabetes, and cancer. Particularly, using Asiatic acid in interstitial cystitis has proven to be successful in rat’s model of cystitis induced by cyclophosphamide, this resulted in the decrease of bladder pressure, detrusor overactivity the normalization of biomarker levels and reduction of bladder edema and thickness of urothelium. The efficacy of Asiatic acid observed at a dose of 30 mg per kg per day for consecutive 14 days was studied in CYP-induced cystitis in rats. After CYP treatment (200 mg/kg, intraperitoneal), Asiatic acid decreased the bladder basal pressure, and overactivity of the detrusor muscles however, it increased the threshold pressure and compliance with the bladder. It also revived the biomarker level of bladder urothelium as well as detrusor muscles while reducing the thickness of urothelium and edema of the bladder. The outcome of this study therefore postures asiatic acid as a powerful and effective remedy for CYP-induced cystitis in rats (Wróbel et al., 2021).

### Coffea arabica

*Coffea arabica* or coffee plant is composed of several essential phytochemicals including polyphenols. Chlorogenic acid is a foremost constituent of coffee plants (Monteiro et al., 2019). The literature reports hepatoprotective, neuroprotective, antimicrobial, and anti-inflammatory effects (Naveed et al., 2018). In a study, ICwas induced in Sprague-Dawley rats by intraperitoneal injection of cyclophosphamide. The test group, treated with chorogenic acid exhibited prominent anti-inflammatory effects. Western blot analysis confirmed that the expression of anti-apoptotic gene Bcl-2 was upheld in the chlorogenic acid-treated group as compared to the control group, while the expression of pro-apoptotic proteins such as Bax and caspases was lower in chlorogenic acid-treated group. Immunohistochemistry analysis promulgated the downregulation of the NFκB pathway in the chlorogenic acid-treated group (Luo et al., 2020).

### Cuminum cyminum

The ethanol was used in the extraction of *Cuminum cyminum* seed to produce the ethanol extract (Jabeen et al., 2017). The pharmacology of thyme extract includes bioactive compounds which are p-menthol, cuminaldehyde, y-terpinene, and beta-pinene. Research has indicated that *C. cyminum* contains extracts that exhibit anti-bacterial, anti-inflammatory, free radical, and pain relief effects, among others due to the phytochemical constituents such as terpenes, phenol and flavonoids (Agarwal et al., 2019; Ramya et al., 2022b; Ramya et al., 2022a). Although there is no specific reference to the use of *C. cyminum* ethanol extract regarding interstitial cystitis in the given contexts, the plant seems to have some properties in the therapeutic management of interstitial cystitis, which is a bladder inflammation-associated condition and is marked by pain. An inflammation illness, interstitial cystitis does not respond well to modern medication. Therefore, this study investigated the aqueous ethanolic extract of the *C. cyminum* effect (AEECC) on cyclophosphamide-provoked bladder toxicity in female rats. Concisely, AEECC at the doses 250 and 500 mg/kg revoked stimulated CYP nociception, bladder weight and serum V.P., edema and hemorrhage, NO, IL-6 and tumor necrotic factor-alpha levels in the sample and showed significant anti-nociceptive and anti-inflammatory results. It was also successful in increasing the activities of the antioxidant enzymes such as catalases and glutathione peroxidases in the beef. AEECC results in a decrease of smooth muscle tone in isolated bladder strips and this was accompanied by a blockade of the effects by several inhibitors. Explorations *in silico* for bioactive compounds revealed antioxidants and anti-inflammatory constituents to support the uroprotective outcome of AEECC (Anjum et al., 2023b).

### Curcuma longa

Curcumin, popularly known as the natural pigment of *Curcuma longa* rhizomes essential for medical findings is universally explored and reported for its anti-inflammatory, anti bacterial activity, antiviral role, antifungal and anti-cancerous activities (Nanjwade et al., 2015; Lee et al., 2013). The compound curcumin suffers from problems such as low solubility and rapid metabolism making bioavailability in people quite low (Urošević et al., 2022). This is because efficient curcumin extraction methods including extraction by soxhlet, ultrasonic, and microwave have been established (Urošević et al., 2022). Similarly, for the qualitative information, electrochemical techniques such as DC polarography and differential pulse polarography have been employed to study the characteristics of curcumin which assist in its detection in natural complex and pharmaceutical preparation (Modi and Pitre, 2010). Given the fact that curcumin demonstrates potential as a treatment in such conditions as dyspepsia, peptic ulcer, and inflammatory diseases more trials are still required to determine its efficacy fully (Asher and Spelman, 2013). Swiss albino rats of the male breed were established with hemorrhagic cystitis through cyclophosphamide administration using intraperitoneal injection with a dose of 150 mg per kg. Generally, the present study has demonstrated that allowing to receive curcumin at the dose of 200 mg per kg intraperitoneal for 10 consecutive days before the injection of cyclophosphamide had significant protective effects. Curcumin reduced Schnorf’s lesions resulting in decreased congestion, edema, and inflammation of bladder tissue. With the help of the remedy WJ-A, it minimized TNF-alpha and nitric oxide, increased ATP and antioxidant levels in the bladder and normalized the ion disorders. Hence, considering the above observations, it can be inferred that curcumin given at a specific dose of 200 mg per kg may be beneficial in regulating cyclophosphamide-induced cystitis by its antidotal activity on inflammation, oxidative stress along with modulating the mechanical properties of bladder smooth muscle tissue (Arafa, 2009).

Curcumol is a sesquiterpenoid found in the medicinal plants of genus *Curcuma*, such as *C. longa* (Sun et al., 2017). *In vitro* studies of curcumol on murine-derived macrophage cell line (RAW264.7) have illustrated promising outcomes in terms of inhibiting the expression of iNOS, TNF-α, IL-6, c-Jun-NH2-terminal kinase (JNK) and other pro-inflammatory proteins (Chen et al., 2014). JNK is a protein that is activated after inflammatory insults leading to maturation, activation and infiltration of immune cells at the sites of inflammation and transcribing proteins such as TNF-α (Naseer et al., 2015). A study investigated the anti-inflammatory role of curcumol in interstitial cystitis. Female mice were administered cyclophosphamide through oral gavage every 3 days to induce interstitial cystitis. Later, the test group was treated with curcumol in doses of 40 mg and 80 mg per kg orally. The bladder samples of curcumol treated mice showed protective effects of curcumol. Cyclophosphamide-induced cellular damage was markedly prevented in the test group. Immunohistochemical analysis showed that levels of TNF- α, IL-1β, and lactate dehydrogenase (LD) were reduced in the curcumol treated mice group (Wu K. et al., 2019). It was also discovered that the levels of protein tyrosine kinase 2 (PTK2) were also suppressed in the curcumol-treated group of mice. PTK2 signaling upholds the transcription and translation of pro-inflammatory signaling molecules majorly TNF-α, IL-1β, and some cell adhesion molecules involved in inflammatory responses (Murphy et al., 2019).

### Egletes viscosa

Ternatin, a flavonoid isolated from *Egletes viscosa*. Therein, this compound has exhibited significant pharmacological efficacy have been noted when the medication has been applied in treating interstitial cystitis. Literature has determined the comparative analysis of ternatin with mesna, the traditional uroprotective compound for ensuring against hemorrhagic cystitis resulting from cyclophosphamide or ifosfamide (Vieira et al., 2004). Altogether the findings of this study indicate that it is possible to replace one or two doses of mesna with ternatin with a positive outcome in terms of prevention of hemorrhagic cystitis thus indicating that ternatin can indeed be therapeutically likely used in the treatment of the condition in question. Furthermore, it was believed that synthetic derivatives of ternatin have shown cytotoxic effects directed only toward cancer cells, moreover, it was determined that it has even higher activity than natural ternatin (Carelli et al., 2015). Overall, the data obtained in this study can be translated into the possible clinical application of ternatin and its derivatives in the management of interstitial cystitis as a new efficient approach. Formulation of mesna supplemented with Ternatin isolated from Egletes viscosa Less in the management of urotoxicity. In experimental HC induced by cyclophosphamide and ifosfamide in male Wistar rats. Few animals one or two of the mesna treatments were substituted by ternatin or 3 mesna doses were substituted with dimethyl sulfoxide. Cystitis was observed for about 24 h after treatment with cyclophosphamide or ifosfamide. Cyclophosphamide or ifosfamide led to substantial macroscopic and microscopic alterations, all of which were notably prevented by either three doses of mesna or Ternatin substituted for either one or two doses of mesna. Nevertheless, these strategies that including replacing two doses with saline, all doses with ternatin, and DMSO failed to intensely reduce HC. Thus, the use of ternatin in substitution of one or two daily doses of mesna fully protected rats from Hemorrhagic cystitis. Mesna is crucial at the first stage of uroprotection (Vieira et al., 2004).

### Epimedium koreanum

The flavonoids present in *Epimedium koreanum* majorly Icariin, be included in active constituents of Epimedium species subgenomes and employ medicinal applications in Chinese traditional therapy (Chen et al., 2011; He et al., 2020). It is mainly derived from *Epimedium grandiflorum* which is through enzymolysis with beta-glycosidase or ethanol extraction then through the step of low-alcohol macroporous resin enrichment and recrystallization. In pharmacological studies, icariin has prospects on neurodegenerative diseases, various cardiovascular diseases, inflammatory aspects, reactive oxidative stress, and tumors (He et al., 2020). Though the employment of Icariin in cystitis in particular according to the given contexts is not mentioned, the broad profile of pharmacologic action thanks to which Icariin has already been successfully applied in various pathologies, shows its potential for application in the treatment of cystitis due to its immunomodulatory activity and preventing accounting for the renal tissue damage, thus, it is should be studied further about the cystitis treatment. Icariin is a prenylated flavonoid obtained from *E. koreanum* used in the protection of cystitis against CYP-induced symptoms in mouse. In mice model single dosage of 150 mg per kg of cyclophosphamide was administered intraperitoneally then different icariin doses were administered intraperitoneally such as 5 mg, 25 mg, and 50 mg per kg were shown to reduce visceral reflexes and nociception. In a chronic model, mice were treated with Icariin 25 mg per kg administered intraperitoneally to 10 days before the initial dose of CYP that is 75 mg per kg given through an intraperitoneal route every 3 days for about 10 days to decrease the edema and hemorrhage of bladder, vascular permeability, and mast cells’ infiltration as well as fibrosis of the lesion tissue. Moreover, Icariin increased antioxidant enzyme levels and activities accompanied by decreased oxidative stress and inflammatory cytokines. On the molecular level, it increased Nrf-2/HO-1 and decreased NF-κB, iNOS, COX-2, and TRPV1, which indicates its further prospectives the extrapolation in the management of CYP-caused cystitis pain (Amanat et al., 2022).

### Eucalyptus phellandra

*Eucalyptus phellandra* is a medicinal plant with expansive biological activities (Miguel et al., 2018). Alpha phellandrene, a cyclic monoterpene, being the principle phytochemical in *E. phellandra*, serves as an anti-inflammatory and scavenging properties (Siqueira et al., 2016; de et al., 2019). Some other medicinal plants also contain Alpha phellandrene including *Eucalyptus staigeriana*, *Schinus molle,* and *Ligusticum marginatum* (Gilles et al., 2010; Bendaoud et al., 2010)*.* In an *in vivo* study, the anti-inflammatory actions of alpha phellandrene were evaluated using mice model. There were many control groups in the experiment including positive and negative controls. The test group was pretreated with various concentrations of alpha phellandrene with a maximum concentration of up to 100 mg/kg. Afterward, cystitis was induced using ifosfamide via the intraperitoneal route (400 mg/kg). Important markers such as myeloperoxidase, IL-1β, TNF-α, nitrates, superoxide dismutase and glutathione were measured in the bladder tissue. Alpha phellandrene significantly decreased the levels of pro-inflammatory cytokines such as TNF-α in the alpha phellandrene treated group. ELISA disclosed the retained levels of glutathione in the alpha phellandrene treated mice group, while opposite results were found in the control groups reinforcing the anti-inflammatory effects of alpha phellandrene in ifosfamide-induced cystitis in mice (Gonçalves et al., 2020).

### Ficus carcica

Leave extract of *Ficus carcica* contain many phytoconstituents including epicatechin, caftaric acid, kaempferol, etc. These phytochemicals have exhibited anti-diabetic, laxative, and antioxidant effects (Vendramin et al., 2021; Teruel-Andreu et al., 2021). In a study, cyclophosphamide-induced induced IC was induced in a female rat model. The animals were divided into six groups including the positive controls and negative controls along with the test group receiving caftaric acid with increasing doses up to 60 mg/kg on the 1^st^, 4^th,^ and 7^th^ day, 1 hour prior to administration of cyclophosphamide. Antioxidant analysis of caftaric acid, vascular leakage of proteins, and quantitative PCR were performed to characterize the antioxidant and anti-inflammatory potential of caftaric acid. A reversal of cystitis was seen in the group pre-treated with caftaric acid. There was no significant increase in bladder weight in the group receiving caftaric acid. The expression of the pro-inflammatory cytokines was significantly retarded in the caftaric acid-treated group, along with restoration of the levels of glutathione and superoxide dismutase. Histopathologically, the hallmarks of edema and urothelial damage were absent for the caftaric acid-treated group (Anjum et al., 2023c).

### Glycine tomentella

*Glycine tomentella* is a traditional Chinese medicinal plant used in immune diseases. Daidzin is an isoflavone that has well-reported anti-inflammatory effects. Mechanistically, these effects are linked with the inhibition of pro-inflammatory protein production in response to LPS-stimulated cells, preventing necrosis in the LPS-affected cells (Tan et al., 2022). These effects of daidzin have also been studied in cyclophosphamide induced cystitis in rat models. In an *in vivo* study, cyclophosphamide-induced IC model was developed in female wistar rats. The first group was co-administered with cyclophosphamide and an extract of *G. tomentella* while the second group was injected a mixture of cyclophosphamide and daidzin. Both the groups were compared with a control group receiving cyclophosphamide + normal saline. The findings of this study displayed a decline in the levels of matrix metalloproteinase-8 (MMP-8), reactive oxygen species, and reactive nitrogen species, hence furnishing antioxidant and anti-inflammatory effects. Attenuation of edema, necrosis, and hemorrhages induced by cyclophosphamide also took place in daidzin and *G. tomentella* extract treated group (Wu KC. et al., 2019). Another study has reported the beneficial therapeutic effects of *G. tomentella* extract in combating the hyperactivity of the bladder in cyclophosphamide-induced IC in rats. The expression of M2 and M3 receptors was downregulated due to the pharmacological actions of daidzin hence relieving the bladder dysfunction in IC (Wu et al., 2018).

### Glycyrrhiza glabra

In the treatment of interstitial cystitis, the compounds of *Glycyrrhiza glabra* extracts include glycyrrhizin that, because of its pharmacologic effects, may be of value in treating the condition. Previous work has shown that glycyrrhizin and its derivative have been established to possess some features such as being anti-inflammatory, anti-viral, and anti-oxidant (Gromova et al., 2022; Wu KC. et al., 2019; Selyutina et al., 2016). Through the above-mentioned experimental analyses, glycyrrhizin has been established to exhibit anti-inflammatory outcomes in the following ways; the suppression of prostaglandin synthesis and the inhibition of cytokine production metformin indications of inflammation as well as the modulation of the activity of immune cells and signaling pathways (Gromova et al., 2022). Also, it could inhibit ROS production, and decrease oxidative stress and strong contraction, fibrosis, and inflammation in cystitis models (Wu KC. et al., 2019). Besides, the roots can also enhance the efficiency of different delivery systems and therefore, boost the bioavailability of such potential therapeutic agents that could potentially change the results of interstitial cystitis treatment (Selyutina et al., 2016). Further, these findings also acknowledge the armour of pharmacologic effects of glycyrrhizin which can be employed for the management of interstitial cystitis. Cyclophosphamide (CP) is a commonly used human or small animal anti-neoplastic agent; however, it is well-known for causing hemorrhagic cystitis (HC). Even though HC can be prevented by Mesna, sometimes, Mesna cannot be enough for all complications. It aimed at assessing glycyrrhizin as an active component of the licorice root Flavonoids on the elimination of CP-associated side effects in fifty-four male adult Sprague Dawley. The control group was administrated with saline and the Cyclophosphamide group was administered with given CP only. The test group consuming coffee procaine (Cyclophosphamide + Mesna) got a daily CP and 3 times Mesna. The groups’ CP plus GLY 100 and CP plus GLY 200 are administered 3 doses of Glycyrrhizin at 100 mg per kg and 200 mg per kg. The Cyclophosphamide + Mesna + GLY100 and CP + Mesna + GLY200 groups had been treated similarly to CP the group but the initial Glycyrrhizin dose was switched by Mesna. The obtained results demonstrated increased bladder preservation in cases with GLY administration in comparison with the CP group, although the effect was not as strong as in the case of Mesna + GLY administration. Such information indicates that GLY could most probably inhibit CP-related HC, and therefore, support Mesna applications (Erdoğan and Keleş, 2019).

### Houttuynia cordata

*Houttuynia cordata*, commonly known as chameleon, contains many important phytoconstituents including alkaloids, glycosides, and terpenoids but flavonoids such as quercetin and hyperoside are the major phytochemicals involved in the biological activity of *H. cordata* extract. Quercetin shows marked anti-inflammatory activity by inhibition of inflammatory signaling pathways such as NF-κB, NLRP3 inflammasome, and cyclooxygenase pathway while hyperoside precipitates anti-inflammatory effects by suppression of NFκB mediated signaling (Azeem et al., 2023; Kim et al., 2011). In an *in vivo* study, the cyclophosphamide-induced ICmodel was developed in female rats. A group of mice were administered with a mixture of cyclophosphamide and normal saline using intraperitoneal injection while the test group was injected with cyclophosphamide and extract of *H. cordata*. The cytokine analysis of the homogenized bladder tissue through enzyme-linked immunosorbant assay (ELISA) showed lower levels of pro-inflammatory cytokines (TNF- α) in the group treated with *H. cordata* extract. The pain threshold was also found to be higher in the test group. Less infiltration of immune cells and absence of hemorrhages was observed in the bladder tissue of extract treated group, showing anti-inflammatory activity of the *H. cordata* extract (Li et al., 2020).

### Ipomoea obscura

*Ipomoea obscura* which is a medicinal plant has been widely researched to show its efficiency in the treatment of interstitial cystitis caused by cyclophosphamide. Solvent extraction followed by fractionation is employed to obtain the plant extract containing phytochemicals, with antioxidant, anti-inflammatory, and antimicrobial activity (Hamsa and Kuttan, 2011). A study conducted in rats proved that the use of an *I. obscura* extract increased the activity of antioxidant enzymes, reduced the extent of tissue damage, and influenced cytokine levels in such a way as to suppress the inflammation related to cyclophosphamide-induced interstitial cystitis (Hamsa and Kuttan, 2011). From these findings, it can be concluded that *I. obscura* has the potential to be used to mitigate the impact of chemotherapy on the urinary system. Another work concerned with the therapeutic potential of the plant Ipomoea obscura against interstitial cystitis in experimental animals that were treated with cyclophosphamide was studied. This study was performed employing Swiss albino mice that received an acute dose of cyclophosphamide (1.5 mmol/kg, body wt, IP) along with an alcoholic extract of *I. obscura* (10 mg/kg body wt ip) for 5 days (Hamsa and Kuttan, 2011).

### Mandevilla velutina

Two compounds, MV8608 and MV8612 from *Mandevilla velutina* were tested for their effects in cystitis using animal models. Both of the compounds produced nearly the same effects when it came to inflammation and hyperalgesia in a rat model of cyclophosphamide induced HC, MV8608 arrested hemorrhage formation and the infiltration of neutrophils while MV8612 reduced the weight of the bladder and the nociception score (Santos et al., 2010). In contrast, Korytov et al. aimed at the treatment of radiation cystitis in rabbits with a gel, containing different compounds that proved the therapeutic effectiveness of the gel regarding clinical and laboratory signs of cystitis. Consequently, the results described in the present paper indicate that both MV8608 and MV8612 from *M. velutina* can be considered as potential candidates to eliminate side effects and control the symptoms of cystitis in the experimental models of the disease and contribute to the elucidation of pharmacological effect of the plants. The reduction of inflammation and relief of pain by the compounds derived from the *Mandevilla veltutina* MV8608, and MV8612 on the model of rats with CYP-induced hemorrhagic cystitis. The male rats were used in the study (6-8 in each group, weight between 220 and 250 g), HC was induced using CYP treatment in the dose of 100 mg/kg by intraperitoneal injection. Nocifensive signs that were used as the behavioral parameters include breathing rate, eye closure, and posture, and these tests were performed at different intervals of time ranging from 15 to 180 min following cystitis. Therefore, markers of inflammation, hematoma, edema, and the increase in bladder weight were analyzed after 24 h of CYP exposure. Neutrophil migration was evaluated from the level of MPO activity at 4 hours following cystitis initiation. Consequently, all these results have shown that the Mesna treatment was capable of decreasing all the inflammatory and the nociceptive values evoked by CYP to a significant extent. Notably, it Another thing that was also realized with it was that MV8608 really reduces hemorrhage formation and also has the potency to reduce the migration of neutrophils Moreover, MV8612, which has a highly strong tendency to decrease the bladder weight and with selfish effect on neutrophil infiltration. Most strikingly, it was found that administration of either MV8608 or MV8612 caused a significant reduction in the nociceptive responses. According to the obtained data, concerning the present study, it is possible to state that MV8608 and MV8612 may bear the demands of the preventative agents against the side effects, particularly the nociception, connected with the usage of CYP in chemotherapy (Santos et al., 2010).

### Matricaria recutita

Apigenin is a key flavonoid and core phytoconstituent of *Matricaria recutita*, also known as chamomile (Abid et al., 2022). Several studies have provided important insights regarding the anti-inflammatory, anti-Alzheimer and anti-cancer activity of apigenin (DeRango-Adem and Blay, 2021; Balez et al., 2016). A study has explored the uroprotective potential of apigenin. Female Sprague−Dawley rats were administered with cyclophosphamide through an intraperitoneal route with a dose of 150 mg per kg at regular intervals on different days of experiments. A group of rats also received oral apigenin an hour before the administration of cyclophosphamide on the same days. Afterward, the rats were sacrificed along with the isolation of the bladder tissue of all the rats. The mRNA was extracted, and a quantitative polymerase chain reaction (qPCR) was performed in order to assess the expression of inflammatory mediators in the bladder tissue of all the rat groups. It was revealed that the levels of mRNA of pro-inflammatory cytokines such as transforming growth factor beta (TGF-β), TNF-α, and interleukin-6 (IL-6) were quite lower in apigenin apigenin-treated rat group as compared to the other group. The Apigenin-treated group also exhibited intact bladder tissue infrastructure as compared to the non-pretreated group. The potential uroprotective and anti-inflammatory activity of apigenin was further confirmed through molecular docking analysis, which provided very optimistic results. Apigenin showed strong affinity with TNF- α receptor for its potential inhibition (Anjum et al., 2023a).

### Moringa oleifera

*Moringa oleifera* raw material has attracted various studies focusing on its most valued part, the leaf, and its method of extraction, and pharmacological profile. The plant contains alkaloids, flavonoids and essential amino acids and hence is used in many therapeutic uses (Darekar et al., 2024; Madukwe et al., 2013). Academic works suggest that chlorophyll contents from *M. oleifera* leaf extracts have hepatoprotective, anti-inflammatory, and antimicrobial traits, that can be used in managing diseases such as cystitis (Aristianti et al., 2021; Jahan et al., 2022). Moreover, the publication also looks into the possibility of using *M. oleifera* extracts in dermatologically acceptable patches to increase its systemic availability and demonstrates a flexible drug delivery system (Chin et al., 2018). Thus, reviewing the vast amount of material on the medicinal properties of Moringa oleifera stated its importance in traditional medicine and its potential for managing numerous diseases, including cystitis. The dose of *M. oleifera* leaves used in this research was fixed to 500 mg per kg per day and 1,000 mg per kg per day through oral route; together with the administration of cyclophosphamide intraperitoneally, 100 mg/kg dose for 7 consecutive days; this evidenced the decrease in malondialdehyde and increase of glutathione (Rojas-Armas et al., 2024; Alrawaiq et al., 2020). Thus, the present investigation demonstrates the cardioprotective role of *M. oleifera* leaves for the oxidative stress due to cyclophosphamide as reflected by the reduction in the levels of malondialdehyde and a rise in the glutathione concentration in the experimental animals.

### Nigella sativa

While *Nigella sativa* (black seed) oil contains the compound **thymoquinone**, pharmacological agents in treating interstitial cystitis: phenoxyacetic acid derivatives that possess selective Beta 3-adrenergic receptors’ (Chancellor and Yoshimura, 2004). Activators, capsaicin-sensitive sensory nerve preventers, oral agents including antihistamines, and tricyclic antidepressants (Whitmore, 2002). These agents seek to treat some of the symptoms that are common in interstitial cystitis such as urinary urgency, frequent urge to urinate, and pain. Although the specific impact of thymoquinone on interstitial cystitis is not described, evaluation of its possible pharmacologic benefits, including administration of inhibition and relieving pain, may be helpful in the situation of finding new treatments for this disease (Amrute and Moldwin, 2007). Cyclophosphamide can cause Hemorrhagic cystitis followed by the events of oxidative stress supported by a low activity of Nrf2. The present study was to some extent meant to establish the extent to which Thymoquinone; a derivable product form *N. sativa* seed extract could alleviate Cyclophosphamide-induced cystitis in mice. Mice were treated with TQ at 5 mg, 10 mg, or 20 mg kg per day intraperitoneally two times daily for 3 days prior and post the 200 mg per kg cyclophosphamide injection. TQ reduced the levels of OS marker and reduced the degree of lipid peroxidation and DNA fragmentation while increasing the activity of antioxidant enzymes and Nrf2 protein level. Histological changes about CYP-induced hemorrhagic cystitis were reduced in treated animals: infiltration of the cells, oedema, and hemorrhage indicated the preventive effect of TQ (Gore et al., 2016).

### Olea europaea

Diverse methods of obtaining oleuropein from its botanical source, *Olea europaea* include percolation extraction with ethyl alcohol (Otero et al., 2020). Extraction with an entrainer such as ethyl alcohol and CO_2_ gas (Cör Andrejč et al., 2022), and extraction by the supercritical method using ethanol and ethyl acetate was done (Soleimanifard et al., 2020). Literature shows anti-oxidant activity, anti-inflammatory effect, anti-bacterial, and anti-viral effects of oleuropein (Otero et al., 2020). Furthermore, the use of microfluidic devices has also been reported to help in the extraction of oleuropein and some of the benefits that come with this include–simplicity, low cost, and eco-friendly (Naleini et al., 2015). Oleuropein, a phenolic compound found in olive leaves or not in the case of side effects of cystitis is a severe condition arising from cyclophosphamide chemotherapeutic. Blood NO, decreased glutathione levels in urine, blood CAT, blood TNF-alpha, VEGF, and blood concentration, and adhesion molecule expression of the gene were analyzed in a rat model after hemorrhagic cystitis. The effects were more severe for the leftist groups for the antioxidants enzymes, glutathione, and catalase while on the rightist for Nitric oxide, it was raised, and the pro-inflammatory cytokines like tumor necrosis factor-alpha, vascular endothelial growth factor. Administration of oleuropein augmented the levels of GSH and CAT while diminution of NO and secretion of tumor necrotic factor-alpha and vascular endothelial growth factor expression. The histological changes in the pattern with the results of the biochemical test supported the uroprotective effect of oleuropein in cases of cyp-induced HC because of its antioxidant activity and anti inflammatory property (Sherif et al., 2016).

### Phyllanthus niruri

Phytochemical characteristics of *Phyllanthus niruri* particularly hydroalcoholic extracts of the leaves discipline flavonoids which contain quercitin, gallic acid, and rutin these compounds have been established to have pharmaceutical value. The plant P. niruri contains phenolic which are powerful antioxidants among them being gallic acid and quercetin (Owabhel and Eboh, 2022; Rusmana et al., 2017). A synthetic work on optimization of extraction parameters indicates that ethanol concentration, extraction time, and sonication amplitude influence the yield of gallic acid and quercetin from *P. niruri* useful for future extraction (Nguang et al., 2018). Also, microwave-assisted extraction was used for drawing out phenolic content from *P. niruri*, the authors mentioned that the plant contains phenolic compounds and it can be considered as a natural antioxidant source (Alara et al., 2024). These show that the hydroalcoholic content extracted from *P. niruri* including quercetin, gallic acid, and rutin possess antioxidant properties that may be helpful in interstitial cystitis. Hydroalcoholic extract of *P. niruri* and its isolated compounds namely, quercetin, rutin, and gallic acid has been worked on mouse model. Hemorrhagic cystitis was induced from the CYP treatment dose of 300 mg per kg of body weight intraperitoneally. While some of the animals had been given Mesna, others were administered with *P. niruri* extract or some of its constituents. These results suggested that TC- *P. niruri* extract and its active components acted significantly and with similar efficacy about Mesna with the reduction of nociception, edema, and hemorrhage in the CYP. On evaluating the anti-inflammatory activity, it was seen that gallic acid and rutin exhibited very good activity whereas quercetin exhibited better antinociceptive activity than that of standard drug. Moreover, the result also infers that extracts of *P. niruri* and some of their components had liver cytochrome P450 –induced lipid peroxidation effects reducing capabilities, this proves that *P. niruri* and few of its compounds have the efficiency to counter some of the side effect caused by CYP (Boeira et al., 2011).

### Potentilla chinensis

A traditional Chinese medications source plant, *Potentilla chinensis*, has been investigated for pharmacological activity. The compounds of *P. chinensis* extract investigated in previous reports include anti-inflammatory, hemostatic, detoxifying, and antipyretic activities (Jung et al., 2016). In particular, as for the medicinal part, the antlers of *P. chinensis* were examined, and the root of the plant was also defined as a perspective to direction further studies, which include extraction and determination of the content of the main substances (Jung et al., 2016). Furthermore, *P. chinensis* extract exhibits a possibility in managing and preventing oxidative stress-associated diseases such as hemorrhagic cystitis by cyclophosphamide with the normalization of the biochemical changes in urothral tissues, detrusor muscle leading to the reduction of DO (Juszczak et al., 2022). This led to the indication that the extract from *P. chinensis* could complement cyclophosphamide-based chemotherapy and has the potential for a pharmacological application, especially in cystitis.

### Pterocarpus marsupium

Pterostilbene a coumarin phytoalexin distributed in various plant species with *Pterocarpus marsupium* on the top, has numerous pharmacological properties like anti-inflammatory, antioxidant, neuroprotection, and anticancer (Tambe et al., 2023; Jain, 2020). The results have indicated that pterostilbene is superior in bioavailability compared to resveratrol and is, therefore, suitable for several biomedical prospects including anticancer activity, anticancer treatment, cardiovascular disorders, and cognition (Jain, 2020; Estrela et al., 2013). Further, the studies have revealed that pterostilbene has promising applications in cancer therapy, especially melanoma, for several reasons and ways, such as it suppresses the synthesis of adrenocorticotropic hormone and induces cancer cell death (Obrador et al., 2021). Although the use of pterostilbene in interstitial cystitis is not described in the relations given above, based on the properties reported in the current literature for the compound, it is possible to conclude that it has certain therapeutic benefits in the development of IT for this disease. The animals selected for the study were 21 male rats that had been distributed equally among 3 groups at random. The control group as well as the CYP group was given 1 mL per kg per day sunflower orally, the Cyclophosphamide + Pterostilbene group (CYP + PteG) received orally 40 mg per kg Pterostilbene dissolved into sunflower oil daily upto 14 days. On day 9 CYP group and Cyclophosphamide + Pterostilbene group were given only dose intraperitoneally, 200 mg per kg Cyclophosphamide dissolved in saline solution, and the control group an intraperitoneal injection of 10 mL/kg of the saline solution. Due to the analyzed protocol samples of the bladder and kidneys’ tissues were taken for the histopathology. This study observed that Pte attenuated the augmentations in the level of CYP-derived malondialdehyde, total oxidative stress (TSO), oxidative stress index (OSI), and apoptosis of renal tissue and elevated the SOD level. It also reduced the increase of total oxidative stress (TSO), oxidative stress index (OSI) and bladder tissues undergo apoptosis. Further, Pte improved histopathologically accompanying CYP-mediated tissue dysfunction in both the kidney and bladder (Kerimoğlu et al., 2023).

### Punica granatum

Ellagic acid is present in the *Punica granatum*. Studies shows pharmacological prospect of ellagic acid, a bioactive compound present in pomegranate and methods of extraction. Studies have confirmed that ellagic acid is thermostable and it exhibits some pharmacological properties include vasorelaxant, anti-oxidant and anti inflammatory (Usta et al., 2013). Research was being carried out at the identification of cytotoxic effects of methanol extracts of seed and peel parts of pomegranate, which contain ellagic acid on cell lines; with different cytotoxicities (Utami et al., 2018). Also, a new admixture of D-Mannose, ellagic acid from Pomegranate extract, prebiotics, and probiotics has been subjected to look for effectiveness to acute cystitis symptoms in a pilot study without most of the cases using antibiotics to relieve symptoms and increase the quality of life. (Pugliese et al., 2020). In addition, a novel extraction procedure using pomegranate peel has been described for the obtaining of ellagic acid of the desired degree of purity essential for industrial uses because of the relatively low price and ease in its large-scale production (Lu and Yuan, 2008). It was found that *P. granatum* has some profound shielding properties against a number of renal and hepatic pathologies in rats. A range of scientific works demonstrates that *P*. *granatum* can inhibit the formation of bladder cancer (Mortada et al., 2020). 2 mL of unfiltered pomegranate juice is given for 14 days with induction of cystitis by giving 150 mg/kg i. p to the rats. These protective effects are due to the action of *P*. *granatum* on the attenuation of oxidative stress indices; malondialdehyde and nitric oxide and facilitated antioxidant active components and enzymes; glutathione and catalase. PJ also influences the regulation of inflammation and apoptosis; such as NF-κB and caspase-3 levels; thus, its nephroprotection/hepatoprotection impact.

### Quercus infectoria

*Quercus infectoria* contains an important phytochemical, isopropyl gallate (IPG) is a compound with pharmacological effects that seem interesting, especially for the curing of cystitis. Based on the derivatives of gallic acid, in the case of IPG, it was seen that IPG has application in the inhibition of inflammation and acts as an anti-oxidant. Hence, the paper recommended the use of IPG as a treatment about hemorrhagic cystitis which is a side effect of ifosfamide (Almeida de Oliveira et al., 2022). Moreover, the process of purification of such bioactive compounds that are found in plants such as IPG involves solvent extraction, where hexane has been confirmed to be a useful solvent to purify the extract from plants such as galangal which contain bioactive compounds similar to those of IPG (Marwati and Winarti, 2007). Moreover, monoterpene isopulegol found in essential oils demonstrates antiradical, anxiolytic, and anti-inflammatory properties, and thus, compounds similar to IPG should be effective in the treatment of cystitis manifestations (Prospero et al., 2019). The anti-inflammatory and antioxidant profile of IPG put this compound in a position where it could be important in the relief of cystitis as well as preventing bladder damage that may be provoked by some drugs. The study shows isopropyl gallate when given 25 mg/kg p. o indicates a remarkable decrease in edema, oxidative stress, and hemorrhage. These results were concluded from ifosfamide-induced hemorrhagic cystitis in a mouse model. It increases antioxidants, decreasing inflammatory markers, indicating its potential use against cystitis (Almeida de Oliveira et al., 2022).

### Silybum marianum

Silymarin is a flavonolignan compound derived from the seeds of the *Silybum marianum* known as the milk thistle and has been used in Europe as well as Asia for the treatment of liver diseases (Lorenzo et al., 2020). A new method has been developed to extract silymarin rather than the conventional extraction techniques like solvent extraction, supercritical-CO2, and ultrasound-aided extraction (Korany et al., 2019). Silymarin has good pharmacological actions like anti-inflammatory effect, anti-oxidant as well and hepatoprotective activity (Lorenzo et al., 2020; Sobeh et al., 2018). Thus, silymarin’s involvement in the decrease of anti anti-inflammation effect might be useful in the mitigation of symptoms linked with cystitis. Additional studies on silymarin and its possible impact on the health of the urinary tract, especially cystitis, will therefore be useful in the effort to establish the other uses of the substance apart from conditions affecting the liver. This study has aimed to evaluate the effect of silymarin on bladder overactivity in a cystitis rat model prepared by CYP. Female Wistar Albino rats received intraperitoneal injections of CYP at a dose of 150 mg per kg or saline and orally with silymarin or its vehicle for 7 days. The effect of CYP on the isolated tissue was tested using the following parameters: rate of spontaneous contractions and cystometric parameters including intercontraction interval. Silymarin actions reduced the frequency of spontaneous contractions, their amplitude, and AUC and also prolonged the intervals between contractions. Also, histopathological evaluation indicated that silymarin had an antagonistic effect on induced bladder inflammation. These studies appear to direct silymarin as a therapeutic agent of CYP-caused bladder overactivity and the dose and regimen of the treatment with silymarin (Eser et al., 2012).

### Solanum lycopersicum

Lycopene, a principle phytoconstituent found in *Solanum lycopersicum*, is separated from employing processes like the ultrasonic-assisted extraction, microwave extraction, and traditional techniques involving the usage of impurity removal agents and dehydrating agents (Periago et al., 2004; Deng et al., 2021; Gallo et al., 2019). It is for these reasons that lycopene has great nutritional as well as pharmaceutical importance, and as such, is of great potential for utilization in both the food and pharmaceutical industries (Periago et al., 2004). Advancements in technology have indicated that lycopene extraction rate could be enhanced, thus solving the problems of higher costs of production and inadequate supply, and thus the profiles and possible pharmaceutical value have been bolstered (Deng et al., 2021). In addition, the extracted tomato’s lycopene may be used for antioxidant action that may be useful in diseases such as cystitis due to the fact that it opposes oxidative strain and inflammation (Gallo et al., 2019). Reduced oxidative stress seems to be related to amelioration of urological diseases, and lycopene is one of the carotenoid antioxidants that proved effective in treating such conditions. In a study conducted on Adult Wister Rats exposed to cyclophosphamide, the lycopene (given at 0.1 and 0.5 mg/kg) had a preventive impact on the rats’ bladders according to Jamshidzadeh et al. (2009) (Jamshidzadeh et al., 2009).

### Tanacetum parthenium

*Tanacetum parthenium L.* is a conventionally used remedy for migraines and various types of headaches. The exploration of the possible mechanisms for relieving headaches has aided in the discovery of the protection and prevention of neuroinflammation in animal models (di Giacomo et al., 2019). Phytochemicals such as flavonoids, phenolic compounds, and terpenes. A sesquiterpene, lactone parthenolide is the most prominent phytochemical in the extract of *T. parthenium.* In an *in vivo* study, the cyclophosphamide-induced IC model was developed in rats. The test group delivered a dose of lactone parthenolide solution subcutaneously before cyclophosphamide injection. The bladder overactivity was assessed in all the groups that showed bladder overactivity to a higher extent in the control group. The bladders of all the groups were isolated after dissection and subjected to histological and cytokine analysis. Western blotting of the bladder samples from the test group revealed lesser expression of NF-ĸB and subsequent reduced production of Cox-2 and other proteins involved in inflammation, showing marked anti-inflammatory activity of lactone parthenolide in IC (Kiuchi et al., 2024).

The present work included rats with hemorrhagic cystitis elicited by Cyclophosphamide to assess the impact of PC extract. Rats had received CYP (HC induction) or saline, then PCE (500 mg/kg orally) or respective vehicle for 14 days. Concerning the normal function of the rat bladder, the values of PCE had not influenced this organ in the least, however, the treatment significantly ameliorated the complexities of CYP-mediated cystitis by reducing inflammation in the urothelium; the levels of the biochemical reactants, including CGRP, TNF-α, IL-6, etc., were nearly normal after PCE treatment. PCE counteracted the impact of CYP on the detrusor muscle (ROCK1, VAChT) while urinary BDNF/NGF alterations alleviated DO in cystometry. Thus, these findings proposed PCE for consideration as a treatment to address HC caused by CYP in patients, based on antioxidative and uroprotective functions (Juszczak et al., 2022).

### Tinospora cordifolia

This is also pertinent to explicate that other natural compounds like resveratrol, and ursolic acid have also been studied in aspects of mitigating hemorrhagic cystitis induced by cyclophosphamide (Keles et al., 2014). *Tinospora cordifolia*, another medicinal plant, has been used to relieve the urotoxic effect of cyclophosphamide through the alteration of Glutathione (GSH) and cytokine levels (Engin et al., 2021).

### Uncaria tomentosa

*Uncaria tomentosa* belongs to the Rubiaceae family, commonly known as “uña de gato” or “cat’s claw” due to its curved hooks (Naseer et al., 2014). Particularly in traditional Peruvian medicine, preparations of *U*. *tomentosa* have been used for the treatment of gastric ulcers, arthritis, viral infections, general inflammatory conditions, urinary tract disease, and cancer (Heitzman et al., 2005). The wide range of activities conferred to *U*. *tomentosa* is mostly attributed to the presence of three main fractions of secondary metabolites: polyphenols, alkaloids, and quinovic acid glycosides. *U*. *tomentosa* decoctions have demonstrated an anti-inflammatory potential by preventing or modulating lung injury induced by ozone *in vivo* (Cisneros et al., 2005). For example, hydroalcoholic extracts of *U*. *tomentosa* and quinovic acid glycoside were able to decrease carrageenan-induced paw edema in mice and rats, respectively (Naseer et al., 2022). Although the mechanisms by which *U*. *tomentosa* exerts anti-inflammatory activity remain unclear, its biological actions are likely related to the modulation of TNF synthesis, via NF-κB inhibition [260].

### Veratrum grandiflorum

Resveratrol, a polyphenolic compound is usually bought from the plant such as *Veratrum grandiflorum* via solvent extraction or solid-phase extraction (Amrute and Moldwin, 2007). The pharmacology study shows that resveratrol has the ability to fight inflammation and also has antioxidant properties that can be helpful in IC (Lukban, 2003). IC is also defined as chronic disorders associated with pain in the pelvic region and regular incidence of the urge to urinate; to the present, there is no existing cohesive treatment plan for this medical condition through the use of drugs (Amrute and Moldwin, 2007). Therefore, the probability for the application of resveratrol as an effective anti-inflammatory for the descend the bladder inflammation in IC patients is viewed as a new perspective of this substance (Ogawa et al., 2018). The current research aimed to establish the efficiency of doses of mesna and 3 resveratrol doses in the treatment of HC, induced by CYP in rats. Therefore, forty-six male rats were chosen, six groups of which received CP; five of which injected the liver with the drug at 150 mg per kg intraperitoneally. It was made of groups that received CP alone, CP combined with doses of 20 mg per kg, 40 mg per kg, or 80 mg per kg resveratrol with CP accompanied by a dose of mesna (30 mg/kg, thrice). That is, a 20 mg per kg dose of resveratrol has been proven to have a good protective effect on bladder injury while a moderate effect was seen at 40 mg/kg and no protective effect was observed with a dose of 80 mg per kg. Contrary, while RES showed significant protection to the groups receiving 20 and 40 mg/kg, its efficacy was surprisingly lower when compared to mesna (Keles et al., 2014).

### Viscum album

*Viscum album* also known as mistletoe is a plant that contains evolutionarily active compounds which include flavonoids and polyphenols in pharmacological application (Parihar et al., 2022; Subhan et al., 2018). These compounds can be isolated by using the ethylic alcohol solution and the obtained extracts possess possible therapeutic properties useful in cancer, cardiovascular diseases, diabetes, and other chronic illnesses (Parihar et al., 2022). In addition, *V. album* extracts resulted in an antitumor effect resulting from the phytochemical compounds such as flavones, viscotoxin, and phenolic acids, for cancer therapy (Hashim et al., 2022). *Viscum album* has quercetin, a flavonoid that possesses anti-inflammatory and antioxidants that might assist in conditions such as cystitis. Thus, cystitis has to be pharmacologically treated by using water-Viscum album extracts containing quercetin; this treatment can amount to anti-inflammatory and antioxidant activities (Hashim et al., 2022). The work aimed at determining the protective measures of an active *V. album* (VA)’s methanolic extract and the quercetin (QE) over Cyclophosphamide (CP)-mediated toxicity in mice. Oral administration for over 10 days, VA given with the dose of 250 mg per kg per day and QE at the dose of 50 mg per kg per day either solely or co-administered with CP, exerted marked cardioprotective, uroprotective, and genoprotective effects. They improved the antioxidant enzyme activity and decreased things such as OSM when supplemented with or without CP. VA and QE equally reduced chromosome abnormalities and aberrant cells in bone marrow implying potentialities in lessening cytogenotoxicity influences of cancer chemotherapy (Chen et al., 2011).

### Zingiber officinale

Gingerol, the main bioactive compound of *Zingiber officinale*, is usually isolated through techniques such as Soxhlet extraction and ultrasonic frequency to increase the extraction rate of the product (Sharma et al., 2023; Rahman, 2022). Pharmacologically, gingerol shows consequential anti-inflammatory and antioxidant activity; it has beneficial effects on various diseases like diabetes, and arthritis including cancer (Sharma et al., 2023; Song et al., 2019). Also, ginger essential oil contains gingerol and other terpenes but alludes to inflammation-reducing and pain-easing effects in animal tests (Vendruscolo et al., 2006). Also, the role of adjunctive medications such as gabapentin, which has analgesic activity, enhances the beneficial results of gingerol in inflammatory diseases for instance interstitial cystitis, by decreasing pain and improving the subjects’ quality of life (Hansen, 2000). This work examines gingerols from *Z. officinale* for IFO-induced hemorrhagic cystitis as an alternative treatment concerning the molecular action. Female mice were selected and distributed into five groups; groups included were the control group, ifosfamide group, ifosfamide plus Mesna, and ifosfamide or gingerol. Moreover, before and after IFO 400 mg per kg, through the intraperitoneal route, mesna with a dose of 80 mg per kg was subcutaneously administrated and gingerols 25 mg per kg was orally administrated. Oxidative stress and bladder inflammation was therefore assessed 12 h after the IFO injection. Therefore, Mesna offered bladder tissue shelter by switching on NF-κB and NrF2 pathways. The gingerols being antioxidants and anti-inflammatory in action result in an increase in the cytokine IL-10, thus offering considerable protection to the urothelium (Ferreira et al., 2023).

**FIGURE 2:**
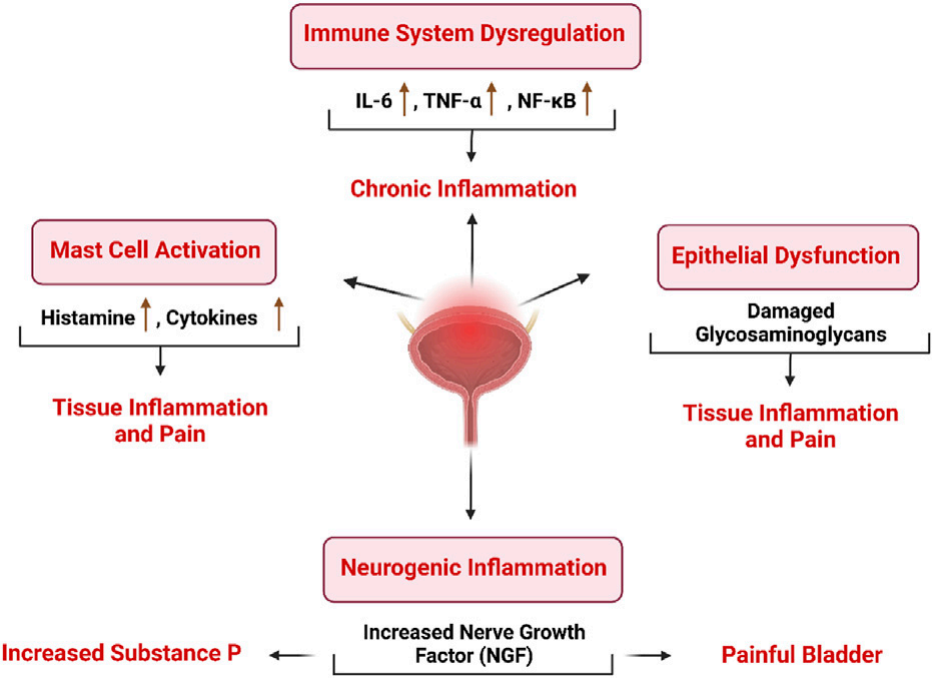
Molecular pathways invoved in intestitial cystitis.

Figure 2 illustrates the molecular pathways involved in IC.

## Urinary-excreted phytochemicals and their protective effects in interstitial cystitis

**FIGURE 3:**
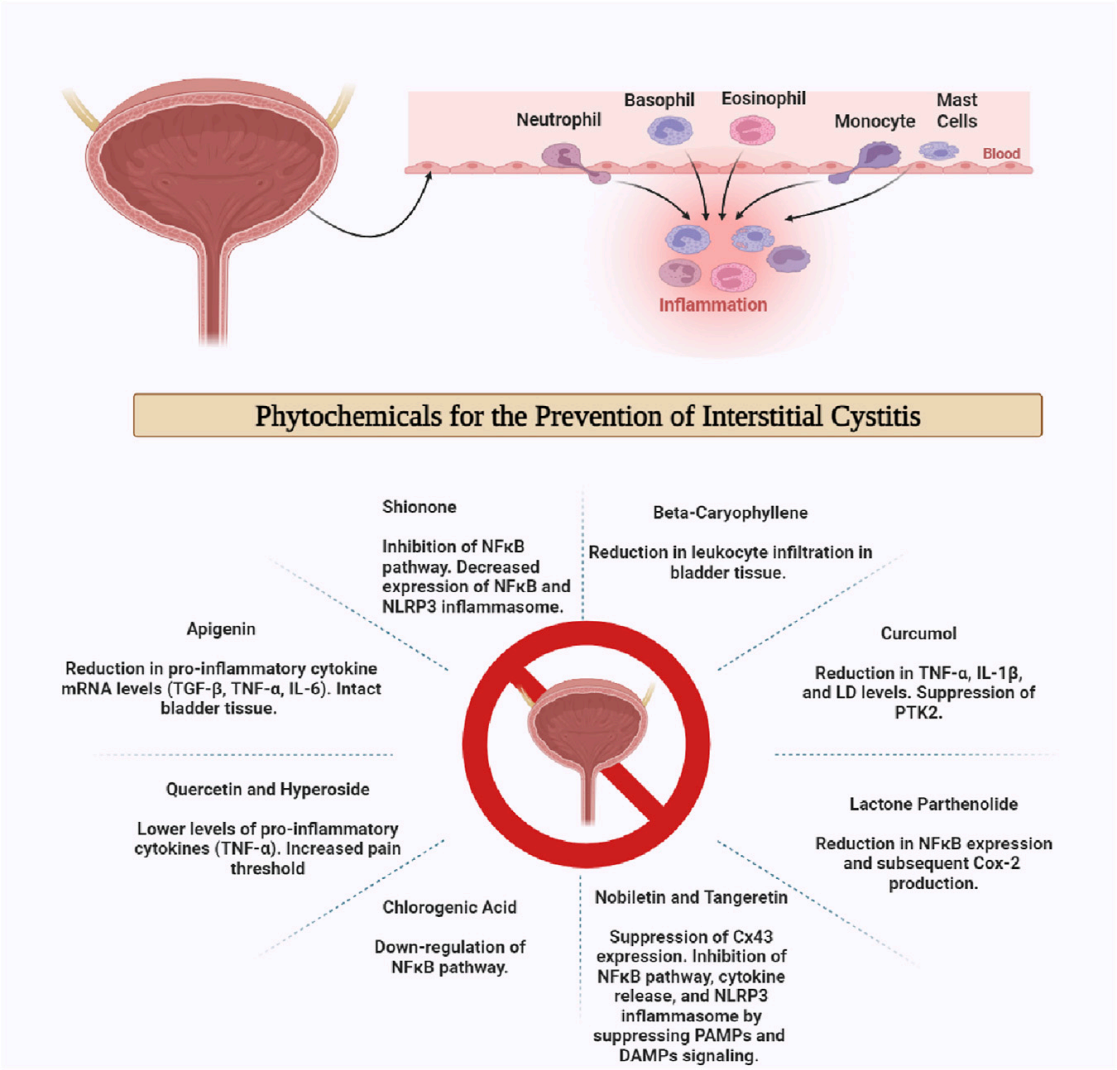
Phytochemicals for the prevention of Interstitial Cystitis.

Several urinary-excreted phytochemicals have been identified for their beneficial effects in interstitial cystitis (IC) management, mainly by reducing inflammation, combating oxidative stress, and strengthening the urothelial barrier. Quercetin, a bioactive compound prevalent in medicinal plants, is recognized for its potent anti-inflammatory and antioxidant effects, aiding in the reduction of cytokine-mediated bladder inflammation. Catechins, derived from green tea, are also eliminated through urine and contribute to bladder protection through antioxidative and anti-inflammatory pathways. Isoflavones, particularly genistein from soybeans, influence immune system modulation and may provide symptomatic relief in IC. Apigenin, found in parsley and chamomile, undergoes metabolic conversion in the liver and is excreted in urine. This process allows its metabolites to act locally in the bladder, reducing inflammation and oxidative damage. Curcumin from turmeric also demonstrates urinary excretion and effectively mitigates chronic bladder inflammation. Resveratrol, a polyphenol from grapes, exhibits anti-inflammatory and tissue-protective properties that are beneficial for bladder health. Epigallocatechin gallate (EGCG), another powerful antioxidant from green tea, protects bladder cells from oxidative stress and suppresses inflammatory pathways. Luteolin, found in bell peppers and oranges, offers anti-inflammatory benefits that support bladder health. Naringenin, predominantly present in lemons and grapefruits, helps reduce oxidative stress and shows protective effects on the urothelial lining. Lastly, ellagic acid, obtained from pomegranates, is metabolized and excreted in urine, where it combats oxidative stress and inflammation in urinary tissues. These phytochemicals highlight the therapeutic potential of dietary interventions in alleviating IC symptoms and enhancing bladder resilience (Yu et al., 2024). The Figure 3 illustrates the potential activity of phytochemicals in attenuating interstitial cystitis.

The pharmacokinetic properties of urinary-excreted phytochemicals play a vital role in their effectiveness in managing IC. Quercetin, despite its relatively good absorption in the gastrointestinal tract, has limited bioavailability due to significant first-pass metabolism. The metabolites are excreted in the urine, allowing it to exert local effects on the bladder. Catechins, such as epigallocatechin gallate (EGCG), undergo extensive metabolism in the liver before being excreted in urine. The absorption of catechins is enhanced when taken with substances like vitamin C, which may improve their therapeutic potential. Isoflavones, including Daidzin, are well-absorbed and metabolized into conjugates in the liver before being excreted through urine, where they contribute to bladder protection. Apigenin, after undergoing hepatic conjugation, is excreted in urine as its sulfated and glucuronidated metabolites, offering high local bioavailability in the bladder. Curcumin, while poorly absorbed in its native form, undergoes significant metabolic transformation in the liver, resulting in metabolites that are excreted in the urine and contribute to its anti-inflammatory effects. Resveratrol, absorbed quickly, undergoes substantial phase I and phase II metabolism, resulting in sulfates and glucuronides that are eliminated through urine. Naringenin, a flavonoid found in citrus, is metabolized and excreted in both free and conjugated forms, showcasing its antioxidant properties. Ellagic acid, after liver metabolism, produces various metabolites that are excreted in the urine, enhancing its anti-inflammatory action. These pharmacokinetic profiles underscore the potential of these phytochemicals to deliver localized therapeutic effects on the urinary system, making them valuable for managing IC. Further research is needed to optimize their dosing and explore the synergistic effects of combined treatments to improve bladder health (Najmanová et al., 2019; Batiha et al., 2020; Chu and Pang, 2018).

## Conclusion and future prospects

Cystitis is a grave condition occurring especially in the patient receiving induction, maintenance, and adjuvant chemotherapy with alkylating agents such as cyclophosphamide and iphosphamide. Many *in vitro* and *in vivo* studies have examined the anti-inflammatory potential of various phytochemicals in the attenuation of IC induced by cyclophosphamide and iphosphamide. *Curcuma longa, Citrus depressa, H. cordata, A. tataricus, G. tomentella*, *B. serrata* and many other medicinal plants contain phytochemicals such as quercetin, curcumol, rutin, isoquercetin, daidzin, boswellic acid, kaempferol, saponins, beta-caryophyllene etc. These phytoconstituents have exhibited potent anti-inflammatory activity in various experiments strengthening the proposition of their potential therapeutic utility in preventing cyclophosphamide/iphosphamide-induced interstitial cystitis. The biological activity of these phytochemicals was comparable and sometimes even more than mesna, in attenuating the inflammatory responses and underlying pathophysiological hallmarks of interstitial cystitis. This review suggests the further replication of these studies to confirm the therapeutic capability of these phytochemicals in providing uroprotective effects. Extensive investigation of the molecular pathogenesis of IC can result in the discovery of many novel targets that may assist in the discovery of new drug molecules, majorly including phytochemicals. Integration of phytochemistry with nanomedicine is also suggested to develop a more targeted and efficacious therapy using phytochemicals to provide uroprotective effects in weakening the inflammatory sequelae in interstitial cystitis.

To further build upon the promising findings from prior studies, future investigations should focus on large-scale, multicenter clinical trials to rigorously assess the therapeutic effectiveness of phytochemicals like quercetin, curcumol, apigenin, boswellic acid and others, in treating IC induced by chemotherapy agents like cyclophosphamide and ifosfamide. Such trials must aim to optimize key factors such as dosage, duration and route of administration to determine the most effective and safe protocols for treatment. Additionally, direct comparisons between these phytochemicals and standard uroprotective agents, such as mesna, would provide critical insights into their relative efficacy. To gain a deeper understanding of their mechanisms, further investigation is necessary to explore that how these compounds mitigate inflammation and oxidative stress, subsequently leading to IC. Phytochemicals like quercetin and curcumol etc. have demonstrated promising anti-inflammatory and antioxidant properties, but further studies are needed to clarify their specific molecular targets, such as their influence on cytokines or their ability to inhibit inflammatory pathways like NF-κB. In addition, utilizing advanced technologies like high-throughput screening, RNA sequencing, and gene-editing techniques such as clustered regularly interspaced short palindromic repeats (CRISPR) could expedite the discovery of new molecular targets for phytochemicals and help identify other bioactive compounds with stronger and more selective effects on bladder tissues. Another promising avenue is the integration of nanomedicine with phytochemical therapies. By employing nanoparticles or liposomal carriers, the bioavailability, stability and targeted delivery of these compounds could be enhanced, ensuring that they reach the bladder tissue in higher concentrations while minimizing systemic side effects. This strategy could significantly improve the therapeutic potential of these natural compounds for patients suffering from IC. Furthermore, long-term studies should be designed to evaluate the chronic impacts of phytochemical interventions on the progression of IC and the quality of life for patients. These studies should monitor recurrence rates, treatment adherence, and patient-reported outcomes to assess the sustainability and overall benefit of phytochemical treatments. Ultimately, the goal would be to establish clinical guidelines based on robust evidence from randomized controlled trials, which could integrate these phytochemicals into standard practice as viable, non-invasive options for managing chemotherapy-induced IC.

This review highlights the unique potential of phytochemicals to advance the treatment of IC, offering opportunities to move beyond traditional therapies. By integrating phytochemical research with cutting-edge innovations such as nanotechnology and molecular targeting, it lays the groundwork for more precise and effective solutions to address chemotherapy-induced IC.

## Abbreviations

ICS, International Continence Society; IC, Interstitial cystitis; PBS, Painful Bladder Syndrome; OAB, Overactive bladder syndrome; UTI, Urinary tract Infection; MC, Mast cell; FIC, Feline Idiopathic Cystitis; GAGs, Glycosaminoglycans; NB, Nanobacteria.
